# Supplementary material for: Designing Accurate Moment Tensor Potentials for Phonon-Related Properties of Crystalline Polymers
Source: Molecules. 2024 Aug 6;29(16):3724. doi: 10.3390/molecules29163724 (PMC11357232; doi:10.3390/molecules29163724)
Supplement: Supplementary file 1 [file molecules-29-03724-s001.zip › molecules-3080880-supplementary.pdf]

molecules

Supplementary Materials

Designing accurate Moment Tensor Potentials for Phonon-related Properties of Crystalline Polymers

Lukas Reicht [1], Lukas Legenstein [1], Sandro Wieser [1,2] and Egbert Zojer [1,*]

[1] Institute of Solid State Physics, NAWI Graz, Graz University of Technology, 8010 Graz, Austria
[2] Institute of Materials Chemistry, TU Wien, 1060 Vienna, Austria
* Correspondence: egbert.zojer@tugraz.at

Table of Contents

S1 Obtaining the Structure of Polythiophene 3
S2 Discussion of Accuracy Measures 3
S3 Influence of Training Data: The Case of PE and PT 5
S4 Influence of the Level: The Case of PE and PT 7
S5 Comparing the Impact of Training Data Generated with an NVT and an NPT Ensemble 8
S6 Splitting Carbon Atoms into Multiple Atom Types 9
S7 Stability Issues During Active Learning for PE 11
S8 Average Atomic Displacement in Molecular Dynamics 13
S9 Ideal Training Data for MTPs Intended for Boltzmann Transport Equation Calculations 13
S10 Elastic Constants: P3HT Calculated with the Clamped Ion Method 15
S11 Elastic Constants: Numerical Problems 16
S12 Elastic Constants: DFT Data 17
S13 Elastic Constants: Mean of Five MTPs 18
S14 Elastic Constants: Effect of Reducing the Plane Wave Cutoff to the Default Value 19
S15 Elastic Constants: MTP Calculation with "Relaxed Ion Method" 20
S16 Elastic Constants: Experiments of PT and P3HT 21
S17 Phonon Band Structure of P3HT over an Extended Frequency Range 22
S18 Comparing Polyethylene Phonon Band Structure with Experiment 22
S19 Band Structures Calculated Using MTPs with Median $RMSD^{phonon}$ 23
S20 Phonon Band Structure of "Best" MTP^{MD} 25
S21 Phonon Band Structures with MTP-Relaxed Unit Cell 25
S22 Phonon Density of States Over the Full Frequency Range 27

S23 Functional Form of the AIREBO Potential 29
S24 Thermal Expansion Calculated with each of the Five MTPs^{MD} of PE 29
S25 Thermal Expansion with Level 18 MTP 30
S26 Thermal Expansion Calculated with the AIREBO Potential 31
S27 Details on Computational Efficiency of MTPs and DFT 32
S28 Details on Computational Efficiency Comparison of MTPs with different Levels 33
S29 Convergence Tests 33
S29.1 Energy Cutoff 33
S29.2 K-Mesh for DFT 34
S29.3 Strain Distance for the Calculation of Elastic Constants 35
S29.4 Supercell Convergence for Calculating Second-Order Force Constants 38
S29.5 Supercell Convergence for Calculating Third-Order Force Constants 39
S29.6 Displacement Amplitude in Phono3py 40
S29.7 Q-Mesh for Phono3py 40
S29.8 Time in Molecular Dynamics Calculation of Thermal Expansion 42

S1 Obtaining the Structure of Polythiophene

As stated already in the main paper, to the best of our knowledge, the crystal structure of polythiophene (PT) has not been unambiguously determined yet. Therefore, we used input from the literature and performed optimizations of the structure with DFT. While we used VASP almost entirely throughout this paper, for this specific task, we used FHI-aims [91], because due to its atom-centered basis set, it is computationally more efficient when unit cells containing a lot of vacuum are considered (see below). Additionally, we typically find FHI-aims to be more reliable in structure optimizations starting far away from the equilibrium, and unit cell angles can be fixed in a straightforward manner, while still optimizing unit cell lengths. For the FHI-aims calculations, we use the PBE functional [43], the MBD-NL correction [92], a system default intermediate basis sets, a 2×3×3 $\boldsymbol{k}$-mesh for the crystal, and we fix the angles to be orthorhombic. As a first step, we construct and relax a single PT chain with a 40 Å vacuum between the chains. This vacuum is large enough that individual chains do not interact with each other. Since a torsional angle was found for 2,2'-bithiophene [93], we set a 40° torsion between the planes of neighboring rings in the starting geometry of the PT chain. Upon relaxation, the PT chain becomes planar, so we conclude that no torsion is present in a PT chain, at least with the employed methodology. Subsequently, we assume an orthorhombic unit cell containing two chains in herring-bone arrangement, as was also performed in previous studies [38,39]. In the starting geometry, the angle between the planes of the two chains is set to ~50°, close to the 55.4° found for the lowest energy structure in a previous study [38]. A small shift along the chain direction (z-direction) is introduced between the chains to break the symmetry of the initial geometry to avoid getting stuck on a saddle point. We set the lattice vector **a** and **b** (perpendicular to the chain) to 1.5 times the value that Zhang et al. used in their simulations [90]. Then, we relax the length of the unit cell vectors and the atomic positions with FHI-aims, whereby the angles are kept fixed. Finally, the unit cell and atomic positions are fully relaxed with VASP, employing the settings described in the main paper. The resulting unit cell compares well with experiments, as can be seen in Table 1 of the main paper. A corresponding structure file is published together with this paper.

S2 Discussion of Accuracy Measures

As discussed in the main paper, there exist multiple possibilities to assess the accuracy of MTPs, when comparing them to the DFT reference data. The considered accuracy measures can be grouped into two categories: those that measure the quality of phonons, and ones that measure the quality of the MTP in an MD setting. In this section, we will focus on the former. To measure the quality of phonons in the main paper, we defined $\mathrm{RMSD}^{\mathrm{phonon}}$ as the root mean square deviation between MTP and DFT phonon frequencies sampled on a dense mesh in the entire Brillouin zone up to 12.5 THz. The cutoff was motivated already in the main manuscript by the relevance of low-energy phonons for quantities like the thermal conductivity. To test the influence of the still arbitrarily chosen "cutoff frequency", we performed tests for polythiophene with a level 22 MTP and training data from 15 K to 500K (i.e., the settings that were used for $\mathrm{MTP}^{\mathrm{MD}}$ in the main paper). Different "cutoff frequencies" of 5 THz, 10 THz, 12.5 THz, and 20 THz are compared in Table S1 for the five MTPs trained in the described way. The table shows that the choice of the "cutoff frequency" has little impact on the relative order of the RMSDs, while their absolute value drops for higher-frequency cutoffs. Most importantly, the best-performing MTP is the same irrespective of the "cutoff frequency", except for the 5 THz "cutoff frequency", where it is second by a very small margin. More generally, the ordering from best to worst stays also almost the same

for all four “cutoff frequencies”. Thus, the “cutoff frequency” is kept fixed to 12.5 THz throughout the main paper to ensure consistency.

Table S1. *Five MTP^{MD} of PT (with different random initializations) are evaluated by the RMSD over the entire Brillouin zone with varying “cutoff frequencies”. The best value in each column is marked in bold. The ordering from best to worst is given in brackets.*

	**RMSD up to 5 THz [THz]**	**RMSD up to 10 THz [THz]**	**RMSD up to 12.5 THz [THz]**	**RMSD up to 20 THz [THz]**
**PT**	0.1000 (#3)	0.0902 (#3)	0.0855 (#3)	0.0795 (#3)
	**0.0870** (#1)	0.0855 (#2)	0.0820 (#2)	0.0774 (#2)
	0.0896 (#2)	**0.0829** (#1)	**0.0804** (#1)	**0.0755** (#1)
	0.1034 (#4)	0.0906 (#4)	0.0881 (#4)	0.1082 (#5)
	0.1397 (#5)	0.1251 (#5)	0.1160 (#5)	0.1019 (#4)
**mean**	0.1039 ± 0.0189	0.0949 ± 0.0154	0.0904 ± 0.0131	0.0885 ± 0.0137

As an alternative to the RMSD over the whole Brillouin zone, one could also compare the performance for Γ-frequencies between the MTPs and DFT. This metric would be cheaper to evaluate, as no supercell calculations are required. The RMSDs for Γ-frequencies for the five MTPs^{MD} of PT are given in the second column of Table S2, while in the first column, the RMSDs over the whole Brillouin zone listed already above (RMSD^{phonon}) are repeated for the sake of comparison. Both metrics yield the same ranking for the independently parametrized MTPs. Thus, we regard the RMSDs of Γ-frequencies also as a valid metric, albeit with somewhat less information content then the RMSD over the whole Brillouin zone.

Table S2. *Frequency RMSDs for five independently parametrized MTPs^{MD} for PT, which are identical to the ones in Table S1. In the first column all phonon frequencies up to 12.5 THz are compared when suitably (see Method section) sampling the first Brillouin zone, the second column considers only Γ-frequencies based on their order, while for the third column, equivalent vibrations are identified via their highest eigenvector overlap (for details see main text). The lowest RMSD values in each column are marked in bold. The ordering from best to worst is given in brackets.*

	**RMSD over whole Brillouin zone up to 12.5 THz (unmatched) [THz]**	**RMSD of Γ-Frequencies up to 12.5 THz (unmatched) [THz]**	**RMSD of matching Γ -Frequencies up to 12.5 THz [THz]**
**PT**	0.0855 (#3)	0.1060 (#3)	0.1140 (#3)
	0.0820 (#2)	0.0950 (#2)	0.1021 (#2)
	**0.0804** (#1)	**0.0928** (#1)	**0.0998** (#1)
	0.0881 (#4)	0.1212 (#4)	0.1304 (#4)
	0.1160 (#5)	0.1308 (#5)	0.1408 (#5)
**mean**	0.0904 ± 0.0131	0.1092 ± 0.0148	0.1174 ± 0.0159

In the above-described comparisons, modes were compared solely based on their order without explicitly considering the associated displacement patterns. As a more elaborate version of the RMSD of Γ-frequencies, we next matched the Γ-modes by the highest overlap of their eigenvectors. Technically, this was performed by taking the dot product of the eigenvectors and identifying the differently calculated modes with highest dot products as “matching modes”. The “Hungarian algorithm” of Kuhn [94] was used to make a unique assignment of modes (see the work of Kamencek et al. [17] for more details). Table S2, in the last column, also contains the RMSDs of Γ-frequencies with eigenmode matching.

By construction, those RMSDs of matching modes are higher than the RMSDs of unmatched modes. In our case, this increase is only rather minor and, more importantly, the ordering of MTPs is not affected by the matching of the eigenmodes.

S3 Influence of Training Data: The Case of PE and PT

In the main paper, we discussed the optimal choice of training data for the case of PE and PT and displayed the distribution of $RMSD^{phonon}$ and $RMSD^{MD}$ values in Figure 4. In the following, the displayed data are listed.

Table S3. *$RMSD^{phonon}$ and $RMSD^{MD}$ (see main paper for definition) of PE with different training data. Training data were generated with MD in an NPT ensemble with the temperatures given in the first column. The level is 22. The lowest value is marked in bold. The standard deviation is given as the "uncertainty" of the mean value. The data correspond to Figure 4a and b of the main paper.*

	**$RMSD^{phonon}$ [THz]**	**$RMSD^{MD}$ [eV/ Å]**
**15 K - 100 K**	0.0730	0.0158
	**0.0720**	**0.0155**
	0.1039	0.0165
	0.1223	0.0159
	0.3130 (outlier)	0.0291 (outlier)
**mean**	0.1368 ± 0.0901	0.0186 ± 0.0053
**15 K - 200 K**	0.1218	0.0131
	0.0806	0.0124
	**0.0739**	0.0129
	0.0948	0.0130
	0.0994	**0.0124**
**mean**	0.0941 ± 0.0167	0.0128 ± 0.0003
**15 K - 300 K**	0.0949	**0.0118**
	**0.0894**	0.0118
	0.1420	0.0120
	0.1222	0.0118
	0.1635	0.0120
**mean**	0.1224 ± 0.028	0.0119 ± 0.0001
**15 K - 400 K**	**0.1031**	0.0113
	0.1084	0.0114
	0.1663	**0.0112**
	0.185	0.0126
	0.1786	0.0158
**mean**	0.1483 ± 0.0353	0.0125 ± 0.0017
**15 K - 500 K**	0.1605	0.0116
	0.1931	0.0112
	0.1817	0.0116
	**0.1517**	**0.0111**
	0.1626	0.0114
**mean**	0.1699 ± 0.0152	0.0114 ± 0.0002

We note that taking the mean value is misleading for the MTP with 15-100 K NPT training data, because there is a particularly strong outlier. The outlier has an $RMSD^{phonon}$ of 0.313 THz, which is much larger than that for the other four MTPs. This gives the wrong impression that the 15-100 K training set produces worse phonons than the 15-200 K or

even the 15-300 K training set. In this case, the median would be a better measure than the arithmetic mean, because it is typically not adversely affected by outliers. While said outlier is particularly strong, also the other training sets occasionally have 1-2 outliers. The presence of outliers demonstrates that it is important to parametrize multiple MTPs and then use a suitable criterion for selecting the best one. Table S4 contains equivalent data for PT. The observed trends are the same as for PE.

Table S4. *RMSD^{phonon} and RMSD^{MD} (see main paper for definition) of PT with different training data. Training data were generated with MD in an NPT ensemble with the temperatures given in the first column. The level is 22. Carbon atoms are split into two types. Best value is marked in bold. Data corresponds to Figure 4a and b of the main paper.*

	**RMSD^{phonon} [THz]**	**RMSD^{MD} [eV/ Å]**
**15 K - 100 K**	**0.0391**	**0.0266**
	0.0427	0.0272
	0.0507	0.0325
	0.0497	0.0290
	0.0487	0.0444
**mean**	0.0462 ± 0.0045	0.032 ± 0.0066
**15 K - 200 K**	0.0686	0.0317
	0.0566	0.0224
	0.0761	0.0253
	0.0551	0.0223
	**0.0547**	**0.0217**
**mean**	0.0622 ± 0.0086	0.0247 ± 0.0037
**15 K - 300 K**	0.0632	0.0227
	**0.0529**	**0.0201**
	0.1191	0.0285
	0.0872	0.0242
	0.0667	0.0207
**mean**	0.0778 ± 0.0235	0.0233 ± 0.0030
**15 K - 400 K**	0.1055	0.0270
	**0.0580**	0.0200
	0.0729	**0.0193**
	0.0753	0.0206
	0.0606	0.0195
**mean**	0.0745 ± 0.0169	0.0213 ± 0.0029
**15 K - 500 K**	0.0855	0.0194
	0.0820	0.0193
	**0.0804**	**0.0192**
	0.0881	0.0288
	0.1160	0.0198
**mean**	0.0904 ± 0.0131	0.0213 ± 0.0038

S4 Influence of the Level: The Case of PE and PT

In the following, we provide tables containing the data plotted in Figure 4c and d.

Table S5. *RMSD^{phonon} and RMSD^{MD} for MTP of PE with different levels. The training data are fixed to 15-100 K NPT. The data correspond to Figure 4c and d.*

	**RMSD^{phonon} [THz]**	**RMSD^{MD} [eV/ Å]**
**level 18**	0.1557	0.0215
	0.1183	0.0241
	0.1988	0.0235
	0.1154	**0.0192**
	**0.1132**	0.0228
**mean**	0.1403 ± 0.0332	0.0223 ± 0.0017
**level 22**	0.0730	0.0158
	**0.0720**	**0.0155**
	0.1039	0.0165
	0.1223	0.0159
	0.3130 (outlier)	0.0291 (outlier)
**mean**	0.1368 ± 0.0901	0.0186 ± 0.0053
**level 26**	0.0543	0.0141
	0.0529	**0.0128**
	0.0436	0.0130
	**0.0433**	0.0138
	0.0603	0.0154
**mean**	0.0509 ± 0.0066	0.0139 ± 0.0009

Table S6. *RMSD^{phonon} and RMSD^{MD} for MTP of PT with different levels. The training data are fixed to 15-100 K NPT. Carbon is split into two atom types.*

	**RMSD^{phonon} [THz]**	**RMSD^{MD} [eV/ Å]**
**level 22**	**0.0391**	**0.0267**
	0.0427	0.0272
	0.0507	0.0325
	0.0497	0.0291
	0.0487	0.0444
**mean**	0.0462 ± 0.0045	0.032 ± 0.0066
**level 26**	0.0303	**0.0207**
	**0.0290**	0.0221
	0.0304	0.0208
	0.0316	0.0217
	0.0404	0.0241
**mean**	0.0323 ± 0.0041	0.0219 ± 0.0012

S5 Comparing the Impact of Training Data Generated with an NVT and an NPT Ensemble

Extending the discussion in section 2.2 of the main manuscript, we tested what influence it makes whether the training data are generated in an NVT ensemble or in an NPT ensemble has. Since in phonon calculations, the unit cell is kept fixed, one might a priori expect that training data from an NVT ensemble are beneficial for phonon calculations, as they more closely resemble the structures that the MTP is used for. To make a comparison between NVT and NPT ensemble training data, we took the NPT ensemble training data that were sampled at 15 K to 100 K, which were considered in the main paper. They contain 129 configurations for PE and 173 configurations for PT. To obtain analogous NVT data, we performed an MD run from scratch with the same settings (except using an NVT ensemble). The NVT data consist of 146 configurations for PE and 179 configurations for PT, which is a similar number as in the case of NPT data. A comparison of the resulting accuracy is shown in Figure S1 in the same style as in the main paper. The corresponding data are listed in Table S7. The MTPs trained on the NPT ensemble data and the NVT ensemble data yield similar $\mathrm{RMSD}^{\mathrm{phonon}}$ values. For PE, the "best" MTP is marginally improved, while the median MTP performs marginally worse, when the NVT ensemble training data are used. For PT, the $\mathrm{RMSD}^{\mathrm{phonon}}$ of the "best" MTP improves by 11 % with the NVT ensemble, which we regard as virtually no change. The median $\mathrm{RMSD}^{\mathrm{phonon}}$ improves by 21 %, which is a slight improvement. The $\mathrm{RMSD}^{\mathrm{MD}}$ of PT comes out unchanged by the choice of ensemble type, while the $\mathrm{RMSD}^{\mathrm{MD}}$ of PE is significantly lower for the NPT ensemble data.

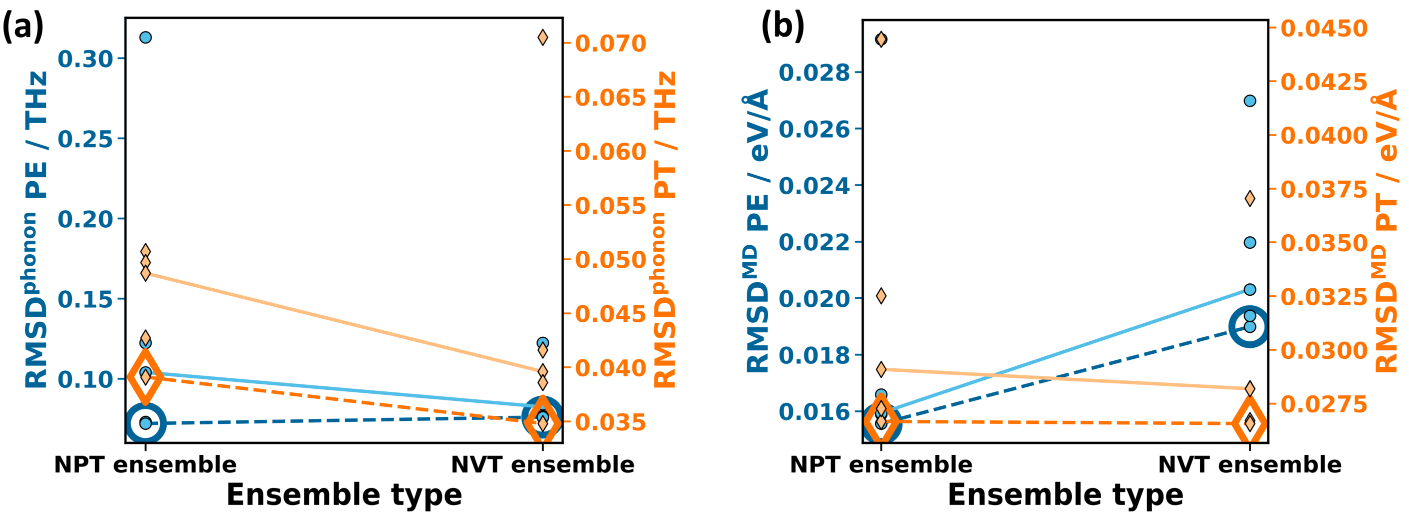

Figure S1. *Influence of the type of ensemble used to create the training data on the accuracy of MTPs. Accuracy is measured by the root mean square deviation (RMSD) between MTP- and DFT-calculated phonon frequencies sampled in the whole Brillouin zone up to 12.5 THz ($\mathrm{RMSD}^{\mathrm{phonon}}$) and by the RMSD of forces on atoms calculated for validation structures generated in a 300 K active learning MD run ($\mathrm{RMSD}^{\mathrm{MD}}$). Panel (a) shows the $\mathrm{RMSD}^{\mathrm{phonon}}$ and panel (b) the $\mathrm{RMSD}^{\mathrm{MD}}$ for PE (blue) and PT (orange). The x-axis denotes whether the training data set is generated in an NPT or NVT ensemble. For each data set, five MTPs are parametrized and the associated RMSD values are shown as small, filled symbols. Datapoints for MTPs with median $\mathrm{RMSD}^{\mathrm{phonon}}$/$\mathrm{RMSD}^{\mathrm{MD}}$ are connected by solid lines, while data for the "best" MTPs are denoted by large, dark, open symbols and are connected by dashed lines. The temperature is set to a temperature ramp from 15 to 100 K. The level has been set to 22, and the carbon atoms of PT are split into two types.*

Table S7. *List of data displayed in Figure S1. Best values for each set of five MTPs are marked in bold.*

	**RMSD^{phonon} [THz]**	**RMSD^{MD} [eV/ Å]**
**NVT, PE**	0.0824	**0.0190**
	0.0766	0.0219
	**0.0760**	0.0193
	0.1223	0.0269
	0.0841	0.0203
**Mean**	0.0883 ± 0.0173	0.0215 ± 0.0029
**NPT, PE**	0.0730	0.0158
	**0.0720**	**0.0155**
	0.1039	0.0165
	0.1223	0.0159
	0.3130 (outlier)	0.0291 (outlier)
**Mean**	0.1368 ± 0.0901	0.0186 ± 0.0053
**NVT, PT**	0.0416	0.0281
	0.0705	0.0370
	**0.0348**	0.0267
	0.0396	**0.0265**
	0.0386	0.0281
**mean**	0.045 ± 0.0129	0.0293 ± 0.0039
**NPT, PT**	**0.0391**	**0.0266**
	0.0427	0.0272
	0.0507	0.0325
	0.0497	0.0290
	0.0487	0.0444
**mean**	0.0462 ± 0.0045	0.032 ± 0.0066

S6 Splitting Carbon Atoms into Multiple Atom Types

In the main paper, we showed that using different atom types for certain atoms in chemically different environments improves the accuracy of the MTP for both phonons, as well as MD forces. Here, we present the corresponding data both for PT as well as P3HT. From the data in Table S8, it can be concluded that splitting the carbons into two types improves the performance of the MTPs across the board. The variation amongst MTPs clearly decreases, as can be concluded from the lower standard deviation, although the latter is characterized by a rather large error due to the few datapoints.

Table S8. *RMSD^{phonon} and RMSD^{MD} for MTPs of PT, where the carbon atoms are treated as one or two atom types. In the latter case, carbons bonded to other carbons and sulfur are distinguished from carbons bonded to other carbons and hydrogen. Training data were generated in an NPT ensemble MD run from 15-500 K. The level of the MTP is 22. The best value is marked in bold. RMSD^{phonon} and RMSD^{MD} are defined in the main paper.*

	**RMSD^{phonon} [THz]**	**RMSD^{MD} [eV/ Å]**
**PT, 1 carbon types**	0.1925	0.0424
	**0.0897**	0.0273
	0.1070	0.0269
	0.1252	**0.0262**
	0.1377	0.0292
**mean**	0.1304 ± 0.0350	0.0304 ± 0.0061
**PT, 2 carbon types**	0.0855	0.0219
	0.0820	0.0218
	**0.0804**	**0.0217**
	0.0881	0.0325
	0.1160	0.0223
**mean**	0.0904 ± 0.0131	0.0241 ± 0.0042

Table S9. *RMSD^{phonon} and RMSD^{MD} for MTPs of P3HT, where the carbons are split into one (i.e., no splitting), three, or six types. Training data were generated in an NPT ensemble MD run from 15-500 K. The level of the MTP is 22. The best value is marked in bold. The data correspond to Figure 5 in the main paper.*

	**RMSD^{phonon} [THz]**	**RMSD^{MD} [eV/Å]**	**Training time [CPUh]**
**P3HT, 1 carbon types**	0.1579	0.0369	
	0.2204	0.0438	
	0.1107	0.0373	
	**0.0901**	**0.0306**	
	0.1246	0.0392	
**mean**	0.1407 ± 0.0455	0.0376 ± 0.0043	1613 ± 295
**P3HT, 3 carbon types**	0.0868	0.0264	
	0.1014	**0.0251**	
	**0.0719**	0.0255	
	0.1062	0.0260	
	0.1042	0.0312	
**mean**	0.0941 ± 0.013	0.0269 ± 0.0022	2602 ± 385
**P3HT, 6 carbon types**	0.0887	0.0237	
	0.0992	0.0237	
	0.2187	0.0297	
	0.0700	**0.0219**	
	**0.0590**	0.0245	
**mean**	0.1071 ± 0.0575	0.0247 ± 0.0027	4323 ± 687

The situation for P3HT (see Table S9) is similar to that for PT. Splitting into three atom types improves the accuracy as compared to no splitting. Further splitting into six atom types does not improve the situation of the average RMSD compared to splitting into three types, because of an outlier for the case of splitting into six atom types. However, the best-performing MTP improves when splitting into six atom types. In Table S9, we also report the average time it takes to train an MTP, which increases when carbon atoms are split into multiple types. Computations are performed on the

VSC-5 supercomputer. It has dual socket AMD EPYC 7713 (Milan) node with 128 cores per node (i.e., 64 cores per socket).

S7 Stability Issues During Active Learning for PE

When performing active learning MD for PE in VASP, we observed that, within the first 1000 time steps, PE chains shift with respect to each other (see Figure S2b). This shift happens during a period where no DFT steps are performed (see Figure S2a). When investigating the trajectories in Ovito [95], it looks like the MTP "relaxes" to this shifted configuration and then stays there. This is, however, a shortcoming of the VASP machine-learned potential (VMLP) trained at that point. As a consequence, the active learning MD run becomes stuck and continues to sample structures in this wrong configuration. This is problematic, as it should instead sample configurations that are close to the real (i.e., DFT) equilibrium structure. The described MD simulations are performed for a 2×3×6 supercell, but the shifts happen simultaneously for all PE chains, such that the shifted structure can be reduced to the primitive unit cell. This makes it easier to visualize (see Figure S2b). To confirm that this shift is merely an artifact of the VMLP, we perform a DFT relaxation starting from the shifted structure, during which it reverts back to the known, non-shifted PE structure. We argue that the root of the problem is associated with the Bayesian error estimation (see blue line in Figure S2a). The error estimation is too low, such that no DFT calculations are performed, while the structure shifts unphysically. To solve the problem, we found two solutions: The first possible solution would be to set the threshold for when DFT calculations are performed (black line in Figure S2a) manually. The second solution would be to set a steeper temperature ramp. The more rapid increase in temperature increases the Bayesian error estimate, which triggers more frequent DFT calculations, preventing the shift. We opt for the first solution and set the initial threshold (ML_CTIFOR keyword) to 0.01 eV/Å for the temperatures 15-30 K, instead of the default 0.002 eV/Å. This has the effect that fewer DFT steps are performed initially (within the first 10 time steps). As a result, the Bayesian error estimation is higher, which, in turn, triggers more DFT calculations later on into the run. These DFT steps prevent the unphysical shift. Instead, the MD run stays close to the real equilibrium.

After this run from 15 K to 30 K finished, it needs to be continued further to higher temperatures, as the training data should eventually be sampled from 15 K to 500 K. Typically, to continue runs, we set the initial threshold to the value at the end of the previous run (0.03 eV/Å in the case of the 15 K - 30 K run). However, in this particular case, when continuing the run, the Bayesian error estimate jumps down to a much lower value, leading to no DFT calculation being performed. Therefore, we again had to set the threshold manually, this time to 0.006 eV/Å as a starting value. With this, we are able to continue the run in a stable manner. To avoid Pulay stress, we split the subsequent runs up into runs from 30 K to 150 K, 150 K to 300 K, 300 K to 400 K, and 400 K to 500 K. As mentioned in the main paper, this strategy is not employed for PT, but for P3HT, where we split the run into segments from 15 K to 300 K, 300 K to 400 K, and 400 K to 500 K.

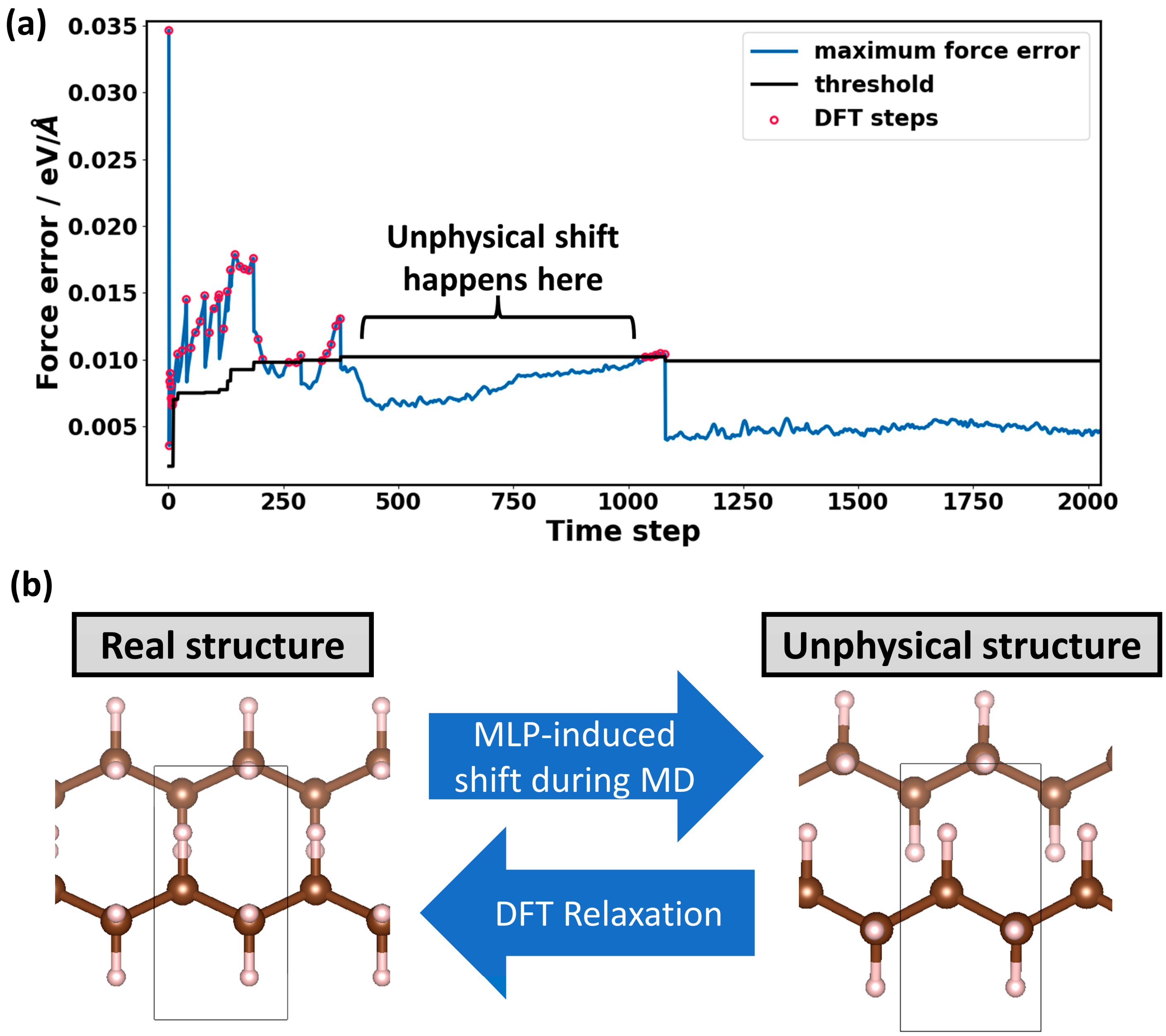

Figure S2. *(a) Maximum Bayesian force error estimate (blue line), threshold (black line), and DFT steps (red empty dots) during an MD run starting from 15 K for PE. Typically, DFT steps are performed when the maximum force error (blue line) exceeds the threshold (black line). An unphysical shift of the PE chains happens during steps 500 through 1000. b) Visualization of the structure with the unphysical shift (right) and of the real equilibrium structure (left). The parametrized VMLP induced an unphysical shift from the real, known structure (left) to the unphysical one (right). This shift happened for multiple independent MD runs and it reverted back upon DFT relaxation.*

For PT and P3HT, no such stability issues occurred. This suggests that this issue is material-specific, and we do not expect it to occur for the majority of crystalline polymers. Nevertheless, this example illustrates that it is absolutely crucial to investigate the atoms' movement during the active learning MD run. This can be performed by loading the XDATCAR (via drag-and-drop) into Ovito [95]. Finally, we want to (again) note that VASP 6.3.0 was used. This is the first version that incorporated VMLPs. Later VASP versions introduced changes to the VMLPs, which could potentially have the effect that the described issue does not occur anymore.

S8 Average Atomic Displacement in Molecular Dynamics

To obtain a rough estimate of the average atomic displacement at finite temperatures, we performed MD runs for PT at 15 K and 300 K in an NPT ensemble with VASP and active learning. The 300 K run was the run that produced the validation set used to calculate $RMSD^{MD}$ (see main paper). For the evaluation, we used a simple in-house script that calculates the atomic displacement from the equilibrium structure at each time step. The mean displacement of all atoms is subtracted to account for a center of mass drift of the whole crystal. Subsequently, the absolute value of the displacement is averaged over all atoms. Its time evolution is shown in Figure S3. As the initial geometry is the equilibrium geometry, the displacement starts at zero and then equilibrates to around 0.6 Å. A 15 K run was performed in the same fashion and yielded an average displacement of around 0.13 Å. This is reflected in forces that are around 4.5 times larger in the 300 K run than in the 15 K run (note that the forces in the spirit of Hooks law are approximately proportional to the displacements, as further discussed in the next section). We note that this only serves as a rough estimate, as we for example did not account for rotations of the whole crystal.

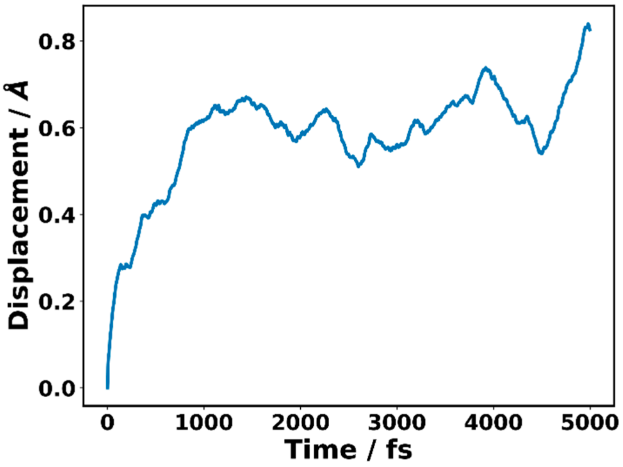

Figure S3. *Absolute value of the average atomic displacement during a 300 K MD run of PT. For details see main text.*

S9 Ideal Training Data for MTPs Intended for Boltzmann Transport Equation Calculations

It is common knowledge in machine learning theory that training data should come from the same distribution as the structures that are seen during application of the machine learning model. The intended applications of the MTP can be grouped into two use cases: The first one is molecular dynamics in a temperature range from 100 K to 300 K. The second one is the calculation of forces close to equilibrium, as is performed to calculate phonons via phonopy and thermal conductivity via phono3py employing the Boltzmann transport equation. For the molecular dynamics use case, it is clear how the training data should look like: since they should be similar to molecular dynamics data, it is best to simply use molecular dynamics data as training data. In our case, we used data from 15 K to 500 K to sample the configuration space well.

The second use case needs more thought, and we will focus on it in the following. Here, the primary goal is to motivate the 15 K - 100 K training data used to parametrize the $MTPs^{phonon}$. To illustrate the nature of the forces in the second use

case, we calculated the norm of forces occurring in phonopy and phono3py calculations and plot their distribution in Figure S4a and Figure S4b. The phonopy calculation (performed to generate the PE phonon band structure) was carried out with the default displacement of 0.01 Å. The phono3py calculation (to obtain phonon lifetimes for PE) was performed with the default displacement of 0.03 Å. In the harmonic case, the force scales linearly with the displacement according to Hook's law. This is reflected in the different distribution of forces of phonopy and phono3py. In phonopy, the maximum force is around 0.5 eV/Å. In phono3py, noteworthy occurrences of forces reach up to 1.5 eV/Å. The observed maximum forces are even around 2.5 eV/Å, but they occur extremely rarely. The maximum (reasonably frequent) force in phono3py of 1.5 eV/Å is three times the value of phonopy, which is expected from Hook's law, as the displacement is three times larger. When an MTP is trained on 15 K data, it can produce excellent phonon band structures, because, for phonopy calculations, the MTP is only required to calculate forces up to 0.5 eV/Å, which is well within the forces it saw during training (Figure S4c). However, when the same MTP is used in phono3py calculations, it predicts thermal conductivities that are only half as large as the DFT reference. This is because, in phono3py calculations, forces up to 1.5 eV/Å are required, which the MTP has never seen in training. The MTP then has to extrapolate from its training distribution, which is a task that is notoriously hard for machine learning methods (at least, extrapolating is certainly much harder than interpolating). In that sense, 15 K MD data are insufficient, if the goal is to perform phono3py calculations. In contrast to that, when we look at the force distribution of 15 K - 100 K MD data (Figure S4d), we see that it incorporates data up to ~3 eV/Å well. Obviously, these forces are not exactly the same as in the phono3py calculations, but it appears that mostly the covered range of forces is relevant. Thus, MTPs that are trained on 15 K - 100 K data can be used well for phono3py calculations, as was demonstrated in the main paper. To conclude, we see that there is a "sweet spot" for what temperatures are ideal to train an MTP for phono3py. On the one hand, 15 K alone is too low. On the other hand, 15 K - 500 K is too high (see main paper). 15 K - 100 K appears to be in the realm of the "sweet spot". We note in passing that we also tested other temperature ranges for the training data. For example, also 15-300 K MD data augmented by additional training data at 15 K worked well for phono3py calculations.

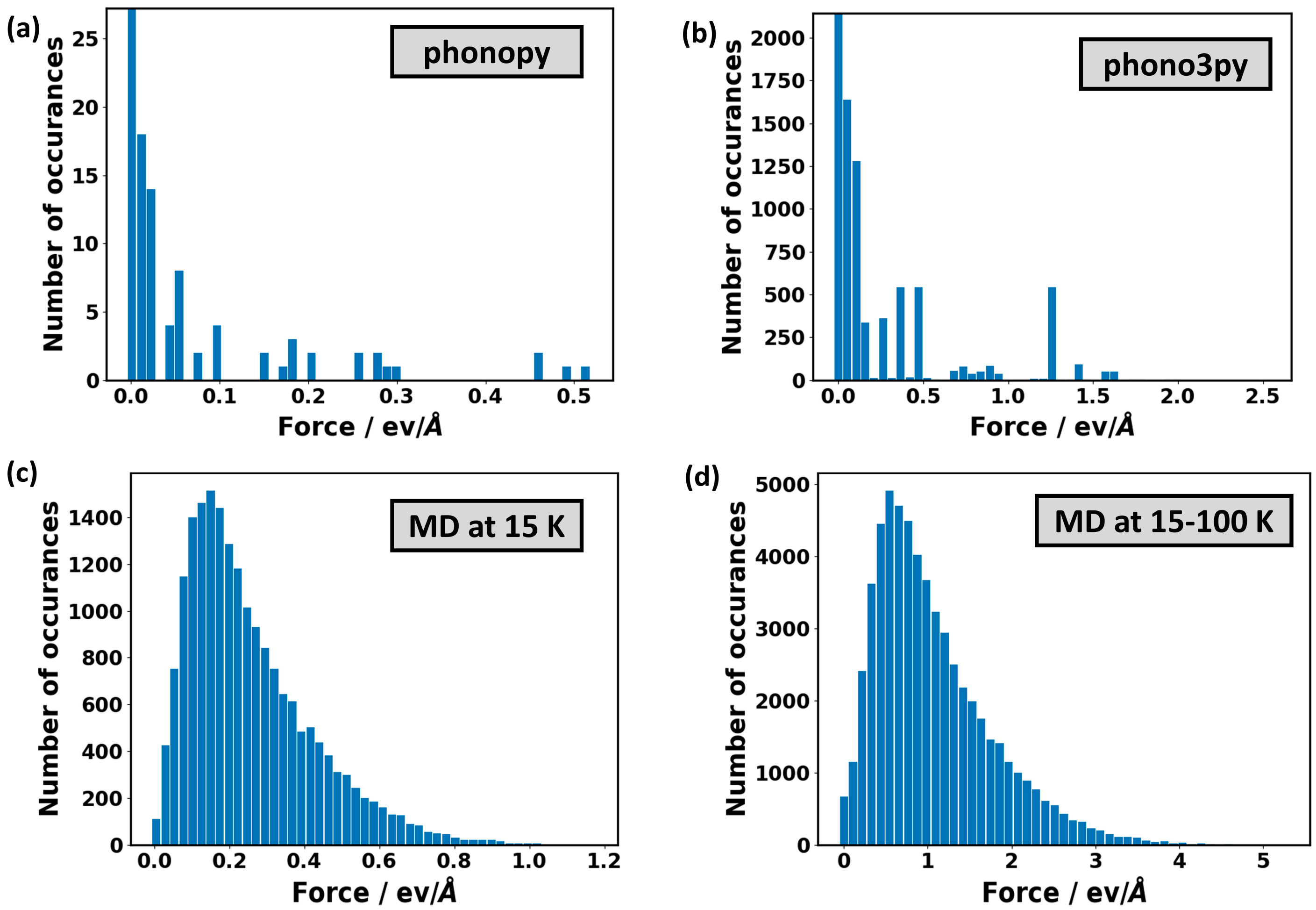

Figure S4. *Force distributions are shown for (a) phonopy and (b) phono3py calculations of PE with DFT, whereby the bar closest to zero is truncated for visibility. For more details see main text. Additionally, force distributions of (c) 15 K and (d) 15 K - 100 K MD runs for PE are shown. Panel (d) represents the training data for* MTP^{phonon} *in the main paper.*

S10 Elastic Constants: P3HT Calculated with the Clamped Ion Method

As mentioned in the main paper, numerical problems occur when calculating the elastic constants of P3HT with the clamped ion method. Still, the resulting comparison between MTP and DFT is shown in Figure S5 to illustrate the issue. To give a numerical example, the elastic constants C_{xx} and C_{yy} will be given, which correspond to how much stress perpendicular to the polymer chain is required to deform the material in that direction. C_{xx} and C_{yy} calculated with the "best" MTP^{MD} deviate by 44 % and 67 % from the DFT reference, when both calculations are performed with the "clamped ion method". When the "relaxed ion method" is instead used in the MTP calculations, this difference drops to 1.1 % (from 44 % for C_{xx}) and to 11.2 % (from 67 % for C_{yy}), thereby showing that there is an issue with the clamped ion method. Notably, performing "relaxed ion" simulations for P3HT using DFT is beyond our computational capacities.

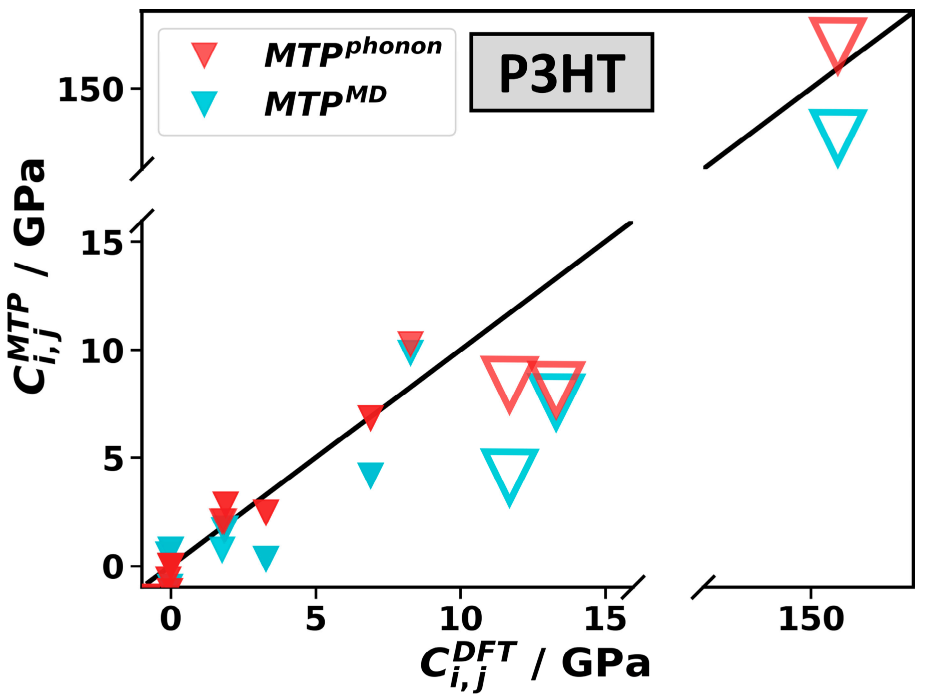

Figure S5. *Independent elastic stiffness tensor elements of P3HT calculated with MTP*^{MD} *and MTP*^{phonon} *compared to the results obtained with DFT. For all calculations, the "clamped-ion method" has been used. The black line with a slope of 1 indicates perfect agreement between MTP and DFT. The displayed data have been obtained with the "best" MTPs as defined at the end of section 2.2. The large open symbols represent* C_{xx}, C_{yy}, *and* C_{zz}, *while the small symbols represent the other independent components of the elastic tensor.*

S11 Elastic Constants: Numerical Problems

In the "clamped-ion method", the elastic tensor is calculated with clamped atomic positions and the contribution from the ionic relaxation is estimated from the second-order force constants. To obtain the final elastic tensor, the contribution from straining the lattice and from the ionic relaxation are subtracted from each other. To give a numerical example, the individual contributions for P3HT are listed in Table S10 and Table S11. It turns out that, especially for the smaller elastic constants of P3HT, the subtraction is numerically problematic, because two large numbers are subtracted from each other to a yield small difference as an elastic constant. For example, to calculate C_{xx} (Voigt notation; equaling XXXX-element in tensor notation), 152.7 GPa is subtracted from 166.3 GPa to yield 13.7 GPa. The situation is even worse for the smallest elements, where the difference between the contribution is sometimes only a few percent of the ion-clamped elastic tensor. This is numerically problematic, because if the calculation of the ion-clamped elastic tensor or that of the estimated ionic relaxation had an uncertainty of a few percent, this could result in huge relative errors, especially when small total elastic constants are obtained. Correspondingly, C_{zz}, the large elastic constant along the long molecular axis, is (relatively) much less affected.

This numerical problem becomes relevant, when either of the two components (ion-clamped elastic tensor or contribution from ionic relaxation) has an uncertainty that is large compared to the final elastic constant. To investigate the respective uncertainties of these components, they are compared between DFT and MTP in Table S10 and Table S11 for P3HT. Focusing on the C_{xx} and C_{yy} elements, we see that the MTP-calculated ion-clamped elastic tensor compares well to DFT, while the contribution from the ionic relaxation has rather large errors when compared to DFT. Therefore, we

attribute the deviations of C_{xx} and C_{yy} between MTP and DFT to the estimation of the ionic relaxation. This is fully consistent with the observation that the clamped ion method and relaxed ion method give different results for P3HT, because the difference in these two methods is how the ionic relaxation is treated.

Table S10. *Ion-clamped elastic tensor of P3HT in GPa. The DFT calculation is given on the left side, while the MTP calculation is given on the right side. Directions (XX, YY, etc.) indicate the tensor element in tensor notation, such that for example the entry in the first row and first column is the XXXX-element, corresponding to* C_{xx} *in Voigt notation.*

	DFT						MTP					
	**XX**	**YY**	**ZZ**	**XY**	**YZ**	**ZX**	**XX**	**YY**	**ZZ**	**XY**	**YZ**	**ZX**
**XX**	166.3	63.4	23.3	-3.9	0	0	167.4	65.0	24.6	-4.2	0.0	0.0
**YY**	63.4	300.3	58.0	-54.6	0	0	65.0	301.4	59.1	-55.4	0.0	0.0
**ZZ**	23.3	58.0	389.0	-17.3	0	0	24.6	59.1	387.7	-17.4	0.0	0.0
**XY**	-3.9	-54.6	-17.3	102.5	0	0	-4.2	-55.4	-17.4	104.0	0.0	0.0
**YZ**	0	0	0	0	154.6	-34.8	0.0	0.0	0.0	0.0	154.6	-35.4
**ZX**	0	0	0	0	-34.8	71.4	0.0	0.0	0.0	0.0	-35.4	72.3

Table S11. *Contributions from ionic relaxation, as estimated from the second-order force constants of P3HT. The units are GPa. The DFT calculation is given on the left side, while the MTP calculation is given on the right side.*

	DFT						MTP					
	**XX**	**YY**	**ZZ**	**XY**	**YZ**	**ZX**	**XX**	**YY**	**ZZ**	**XY**	**YZ**	**ZX**
**XX**	152.7	59.8	21.1	-3.9	0	0	159.2	62.6	21.8	-2.9	0.0	0.0
**YY**	59.8	287.8	50.6	-54.4	0	0	62.6	293.0	52.2	-54.8	0.0	0.0
**ZZ**	21.1	50.6	237.2	-17.2	0	0	21.8	52.2	235.7	-17.3	0.0	0.0
**XY**	-3.9	-54.4	-17.2	100.6	0	0	-2.9	-54.8	-17.3	101.9	0.0	0.0
**YZ**	0	0	0	0	146.1	-34.0	0.0	0.0	0.0	0.0	144.3	-34.0
**ZX**	0	0	0	0	-34.0	69.5	0.0	0.0	0.0	0.0	-34.0	70.2

S12 Elastic Constants: DFT Data

In the following, we list all elastic tensors, which are calculated with the DFT settings described in the Methods section using the clamped-ion method. Additionally, a calculation of the elastic constants of P3HT was performed with the VASP-internal routine and an increased energy cutoff of 900 eV.

Table S12. *Elastic constants of PE calculated with DFT. Units are GPa.*

	**XX**	**YY**	**ZZ**	**XY**	**YZ**	**ZX**
**XX**	12.2	5.2	-0.3	0	0	0
**YY**	5.2	11.4	1.4	0	0	0
**ZZ**	-0.3	1.4	327.8	0	0	0
**XY**	0	0	0	6.2	0	0
**YZ**	0	0	0	0	2.8	0
**ZX**	0	0	0	0	0	1.8

Table S13. *Elastic constants of PT calculated with DFT. Units are GPa.*

	XX	YY	ZZ	XY	YZ	ZX
**XX**	13.1	9.7	-1.1	0	0	0
**YY**	9.7	16.0	-0.7	0	0	0
**ZZ**	-1.1	-0.7	381	0	0	0
**XY**	0	0	0	7.0	0	0
**YZ**	0	0	0	0	1.5	0
**ZX**	0	0	0	0	0	1.6

Table S14. *Elastic constants of P3HT calculated with DFT. The settings are described in Section S3 of the main paper and include an energy cutoff of 700 eV. This calculation is used as reference throughout the main paper and Supplementary Materials. Units are GPa.*

	XX	YY	ZZ	XY	YZ	ZX
**XX**	13.3	3.3	1.9	0	0	0
**YY**	3.3	11.7	6.9	-0.1	0	0
**ZZ**	1.9	6.9	150.9	0	0	0
**XY**	0	-0.1	0	1.8	0	0
**YZ**	0	0	0	0	8.3	-0.8
**ZX**	0	0	0	0	-0.8	1.8

Table S15. *Elastic constants of P3HT calculated with the VASP-internal routine and an energy cutoff of 900 eV. Units are GPa.*

	XX	YY	ZZ	XY	YZ	ZX
**XX**	14.2	3.9	2.3	0	0	0
**YY**	3.9	12.8	7.5	-0.1	0	0
**ZZ**	7.5	7.5	152.2	-0.1	0	0
**XY**	0	-0.1	-0.1	2.0	0	0
**YZ**	0	0	0	0	8.6	-0.8
**ZX**	0	0	0	0	-0.8	2.0

S13 Elastic Constants: Mean of Five MTPs

The elastic constants of the "best" MTPs are reported above and in the main manuscript. In Figure S6, we report the arithmetic means of the elastic constants calculated with five MTPs. Additionally, error bars are provided, which correspond to the standard deviation of said elastic constants.

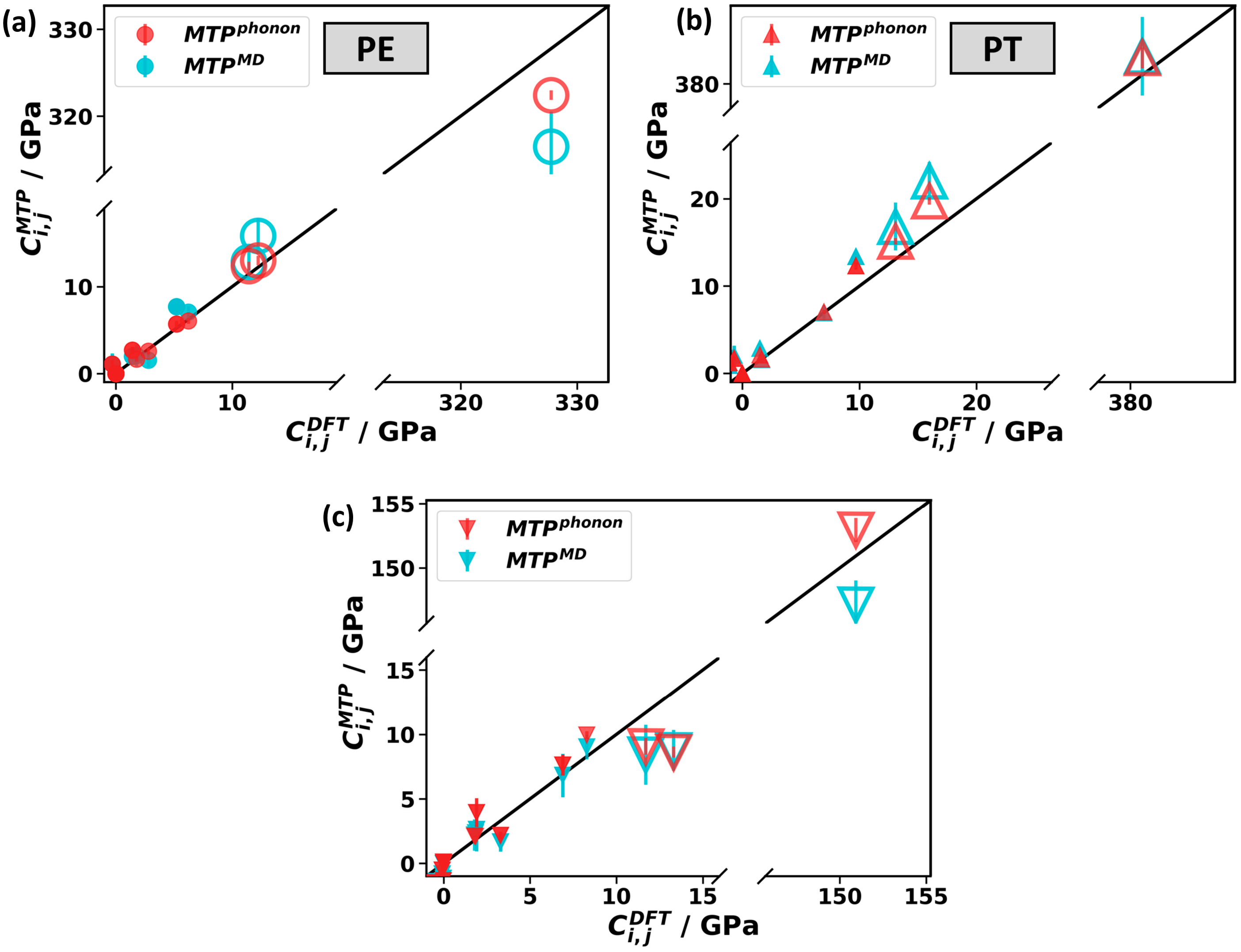

Figure S6. *Elastic constants calculated with* MTP^{MD} *and* MTP^{phonon} *are compared to the DFT reference data. The investigated materials are (a) PE, (b) PT, and (c) P3HT. The black line indicates perfect agreement between MTP and DFT. Datapoints are the mean over elastic constants calculated with (typically five) MTPs, and error bars are the standard deviation of these elastic constants. For* MTP^{phonon} *of P3HT only two MTPs were parametrized due to the high computational cost associated with it. The large open symbols represent* C_{xx}, C_{yy}, *and* C_{zz},*, while the small symbols represent the other independent components of the elastic tensor.*

S14 Elastic Constants: Effect of Reducing the Plane Wave Cutoff to the Default Value

To put the differences between MTP- and DFT-calculated elastic constants into perspective, we also calculated the elastic constants with a lower energy cutoff, shown in Figure S7. For this calculation. we took the default energy cutoff of VASP for the atoms constituting the studied materials. It amounted to 400 eV, whereas we used 900 eV (PE and P3HT) and 700 eV (PT) in the reference DFT calculations above and in the main manuscript. Starting from the structure that was relaxed with a high energy cutoff, the unit cell and atomic positions were relaxed with 400 eV. This relaxation caused rather large unit cell changes, which led to Pulay stress. Therefore, starting from the resulting structure of the first

relaxation, we performed a second relaxation with the same settings. For the so-obtained structure we calculated the second-order force constants and elastic constants with an energy cutoff of 400 eV.

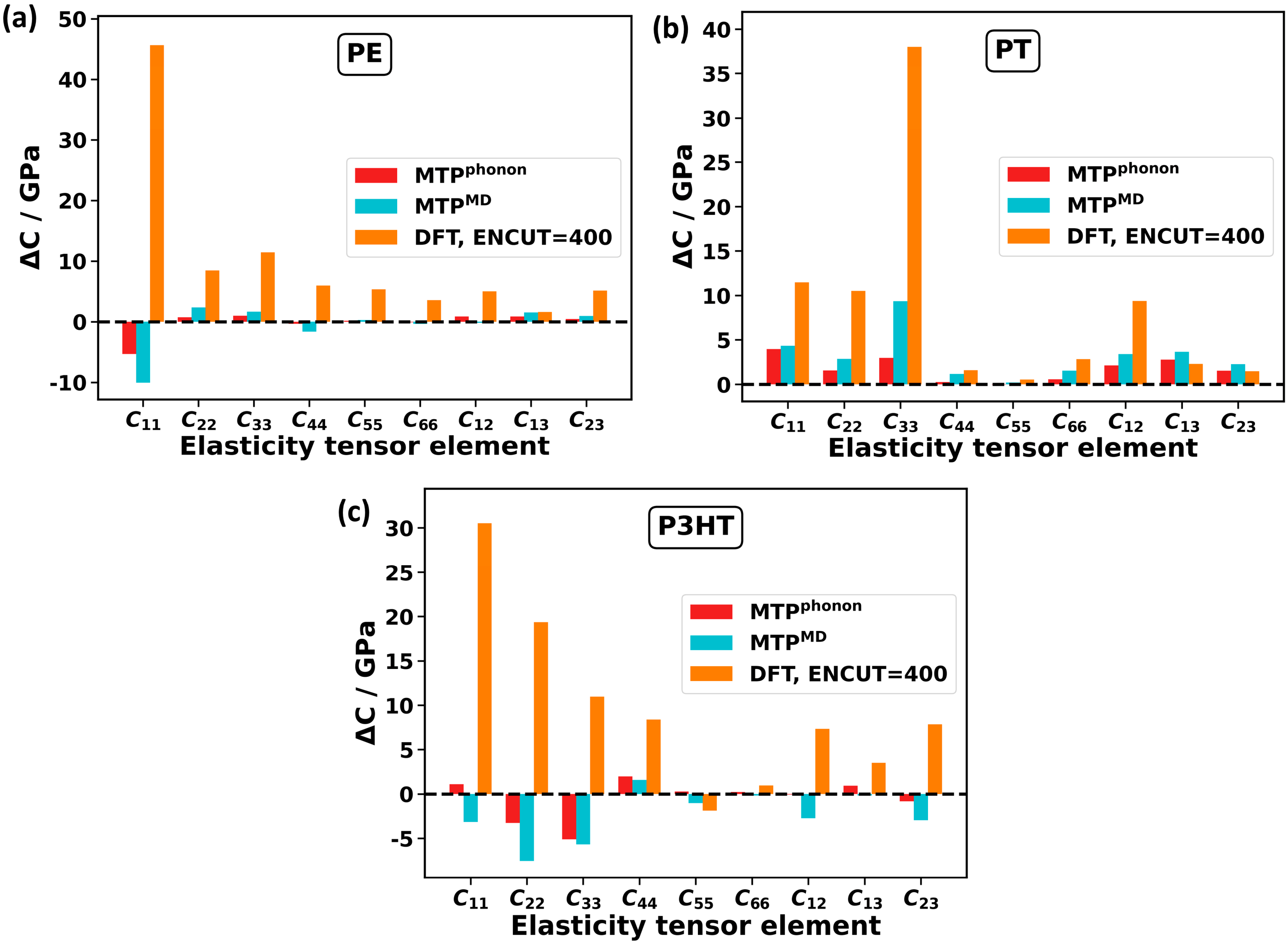

Figure S7. *Elastic tensor elements calculated with MTP*^{phonon}*, MTP*^{MD} *and DFT with energy cutoff 400 eV are compared to the reference DFT calculation with energy cutoff 900 eV (PE and P3HT) and 700 eV (PT). ΔC is the difference between the respective method and the DFT reference. The shown MTP*^{phonon} *and MTP*^{MD} *results are obtained with the "best" MTPs, which have lowest RMSD*^{phonon}*/RMSD*^{MD}*. For PE and PT all independent elasticity tensor elements are shown, while P3HT due to the reduced symmetry has additional very small elements that are not shown.*

S15 Elastic Constants: MTP Calculation with "Relaxed Ion Method"

In Section S12 and the other sections above, elastic constants are calculated with the "clamped ion method" with MTPs and DFT (see Method section of main paper for further details). With the MTPs, we also calculate elastic constants with the less approximative "relaxed ion method", shown in Figure S8. As DFT reference values, we still need to take the "clamped ion method" calculations, because the DFT calculations with the "relaxed ion method" would be computationally too expensive.

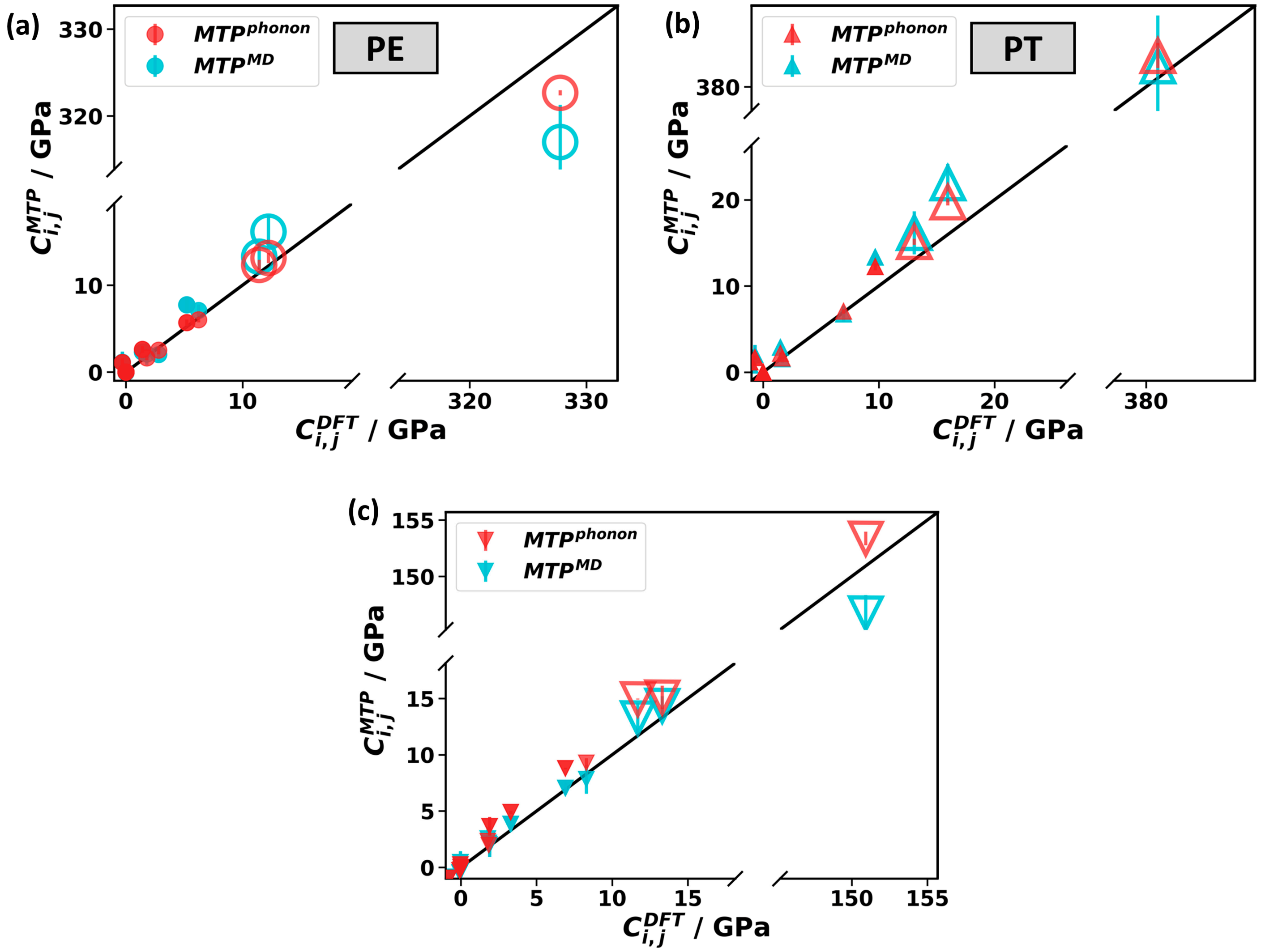

Figure S8. *Elastic constants calculated with MTP^{MD} and MTP^{phonon} are compared against the DFT reference. The investigated materials are (a) PE, (b) PT, and (c) P3HT. The black line indicates perfect agreement between MTP and DFT. Datapoints are the mean over elastic constants calculated with (typically five) MTPs, and error bars are the standard deviation of these elastic constants. For MTP^{phonon} of P3HT only two MTPs were parametrized due to the high computational cost associated with it. In contrast to the analogous Figure 6 in the main paper and in section S12, where the "clamped ion method" was used for the MTPs, we here employed the less approximative "relaxed ion method" for the MTPs. The DFT calculations were still performed with the "clamped ion method" and are identical to those used in Figure 6 and in section S12.*

S16 Elastic Constants: Experiments of PT and P3HT

The literature on experimental elastic constants for PT and P3HT is sparser than that for PE. According to Root et al., typical tensile moduli of P3HT range from 0.1 GPa to 1 GPa depending on the sample and, given its glass transition temperature near room temperature of 12 °C, the temperature at which the measurement was taken [65]. Very recently, the Young's modulus of polythiophene nanofibers was measured to be 64.5 ± 5.0 GPa, which was reported to be an order of magnitude higher than its bulk value [66]. Yang et al. attribute the high Young's modulus of PT nanofibers to the alignment of the molecular chain [66]. This suggests that typically reported values (like 0.1 GPa -1 GPa for P3HT) were measured on samples that are far from (single) crystalline. Therefore, we cannot compare our values obtained for perfect P3HT crystals with experiments. Regarding the 64.5 ± 5.0 GPa measurement of PT by Yang et al., it is not

specified, to which direction their measurement corresponds. Their value lies between our calculated values of 15.6 GPa, 10.9 GPa, and 382 GPa for C_{xx}, C_{yy}, and C_{zz} of PT. Presumably, 64.5 GPa is close to the in-chain direction, but also, in this case, the experimentally studied nanofibers are presumably far from the perfect single crystals considered in the simulations.

S17 Phonon Band Structure of P3HT over an Extended Frequency Range

In the main paper, the phonon band structure of P3HT calculated with DFT and the "best" MTP^{Phonon} is shown from 0 to 9 THz. Here, we show the same band structure for a frequency range twice as large.

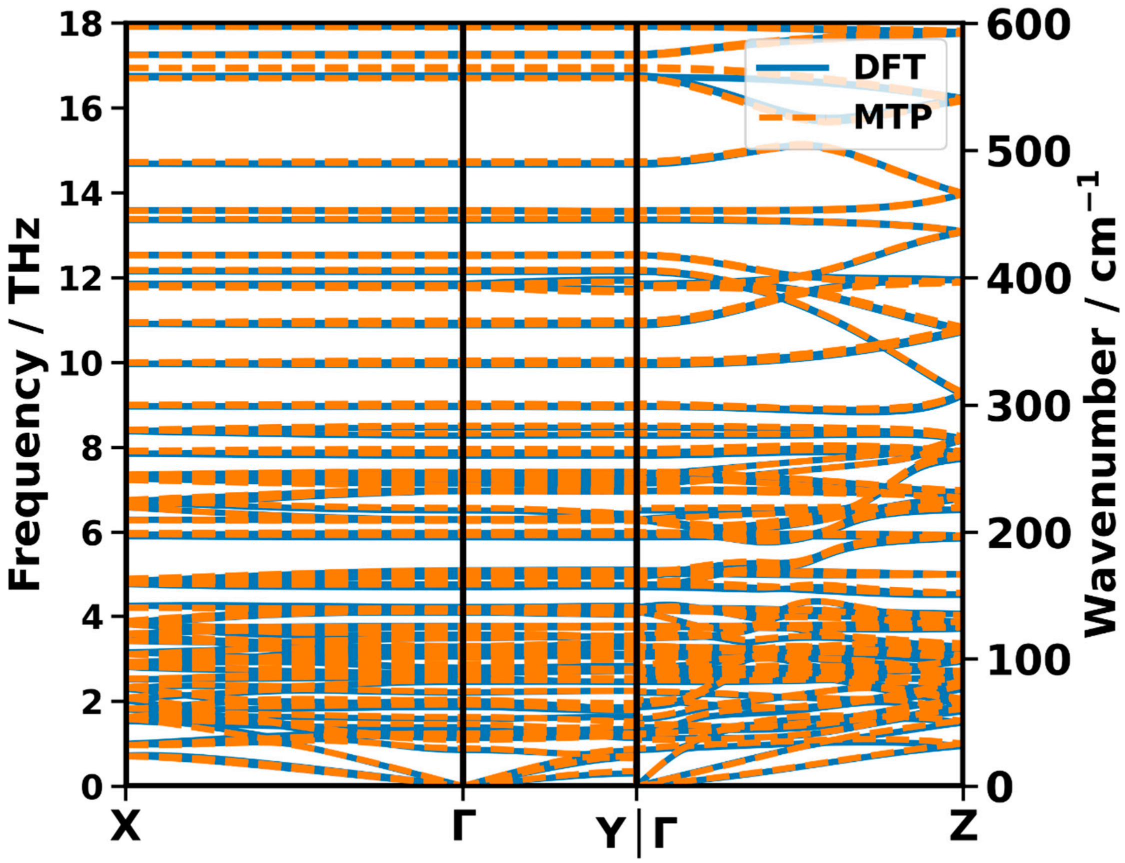

Figure S9. *Phonon band structure of P3HT calculated with DFT and the "best" MTP^{phonon}.*

S18 Comparing Polyethylene Phonon Band Structure with Experiment

The aim of the present paper is to evaluate the accuracy of MTPs by comparing them to DFT data. In the best case, MTPs give the same result as DFT would give. This is, for example, the case for the phonon band structure of PE, as can be seen in the main paper. To complement this, the DFT-calculated phonon band structure of PE is compared to experiments in Figure S10. Schaufele et al. performed Raman spectroscopy on polyethylene molecules with up to 94 carbon atoms [96]. From the overtones of the longitudinal acoustic mode, they deduce the shown data points. These fit very well to our calculated phonon band structure, except for the two points with the largest wave vector. We suspect that this might be a consequence of Schaufele et al. measuring molecules instead of polymeric PE crystals. Feldkamp et al. performed coherent neutron scattering on a deuterated PE crystal with approximately parallel chains, which was

obtained by stretching polycrystalline PE [97]. Their measurements also agree very well with our calculations. This good agreement between the simulations and experiment together with the literature [17,21,82,83] mentioned in the main paper shows that our chosen DFT method is appropriate for PE.

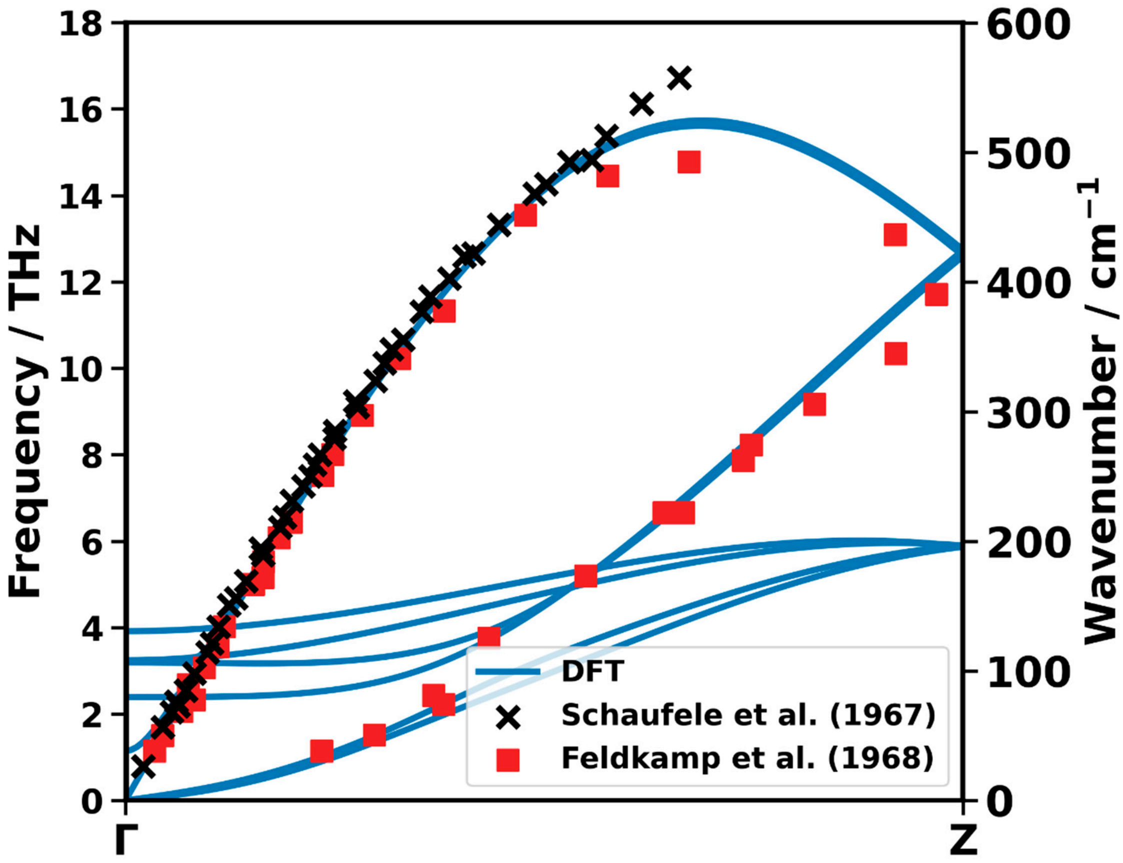

Figure S10. *Phonon band structure of PE calculated with DFT shown with experiments by Schaufele et al. [96] and Feldkamp et al. [97]. Some data points of Schaufele et al. were removed in regions where there are very many points to increase the clarity of the plot.*

S19 Band Structures Calculated Using MTPs with Median RMSD^{phonon}

In the main paper, we reported the band structures with MTPs (following the MTP^{phonon} paradigm) that had the best RMSD^{phonon} (out of five MTPs). Here we report analogous plots, but use the MTPs that have median RMSD^{phonon} (Figure S11 and Figure S12). By "median RMSD", we mean that, out of five MTPs, two perform better and two perform worse. RMSD^{phonon} of the median well performing MTP are 0.054 THz (PE) and 0.032 THz (PT), which is slightly higher than the 0.043 THz (PE) and 0.029 THz (PT) of the best-performing MTP presented in the main paper. This marginally increased RMSD^{phonon} is reflected in the phonon band structures: phonon band structures of MTPs with the median RMSD^{phonon} are slightly less accurate compared to those with the best RMSD^{phonon}, but still show an excellent agreement with the DFT results. For PE, the lowest two bands along the Γ-Y path slightly deviate from DFT (Figure S11). This deviation is not present for the best-performing MTP. For PT, the median well-performing MTP produces a band structure that is hard to distinguish from the band structure of the best performing MTP. For P3HT, there is no "median well performing" MTP, as we only parametrized two MTPs, because of the high computational cost of the parametrization.

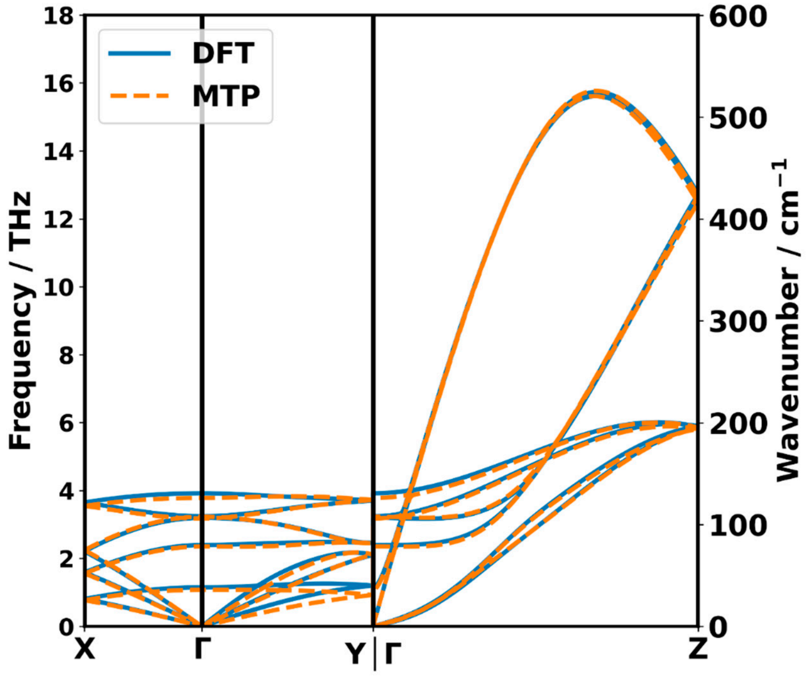

Figure S11. *Phonon band structure of PE calculated with DFT (solid blue line) and MTP^{phonon} (dashed orange line) with median RMSD^{phonon}.*

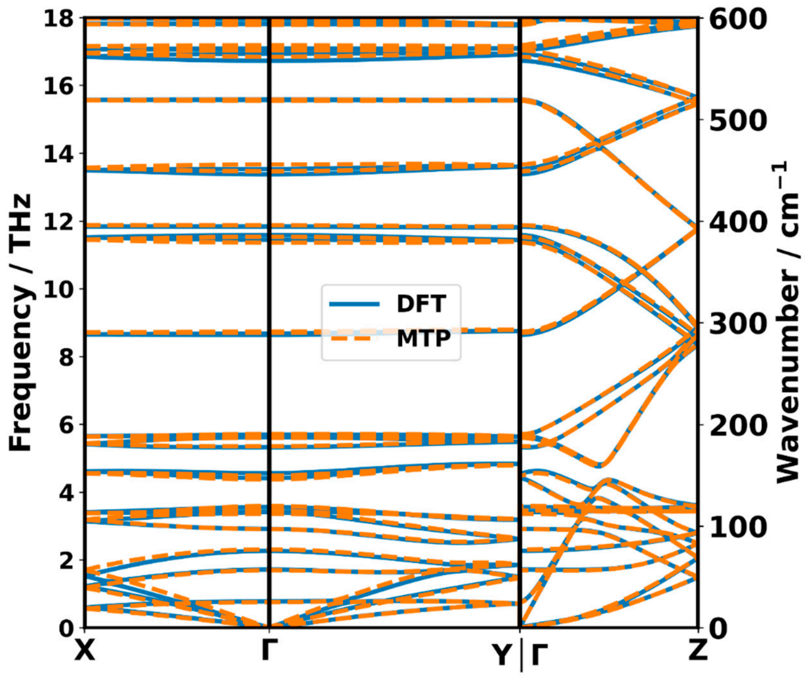

Figure S12. *Phonon band structure of PT calculated with DFT (solid blue line) and MTP^{phonon} (dashed orange line) with median RMSD^{phonon}.*

S20 Phonon Band Structure of "Best" MTP^{MD}

The phonon band structure calculated with the "best" MTP^{MD} for PE is shown in Figure S13. By definition, this MTP has the lowest $RMSD^{MD}$ (and coincidentally also the lowest $RMSD^{phonon}$ amongst the $MTPs^{MD}$). It has two imaginary modes at the Y-point, and also a dip into the negative region along the G→Z path. This is somewhat surprising, considering how well the rest of the phonon band structure matches the DFT reference. Increasing the unit cell size and relaxing the unit cell with the MTP did not resolve the imaginary modes at the Y-point. In fact, it had hardly any impact on the band structure. We note that two out of the five $MTPs^{MD}$ of PE produce phonon band structures with imaginary modes.

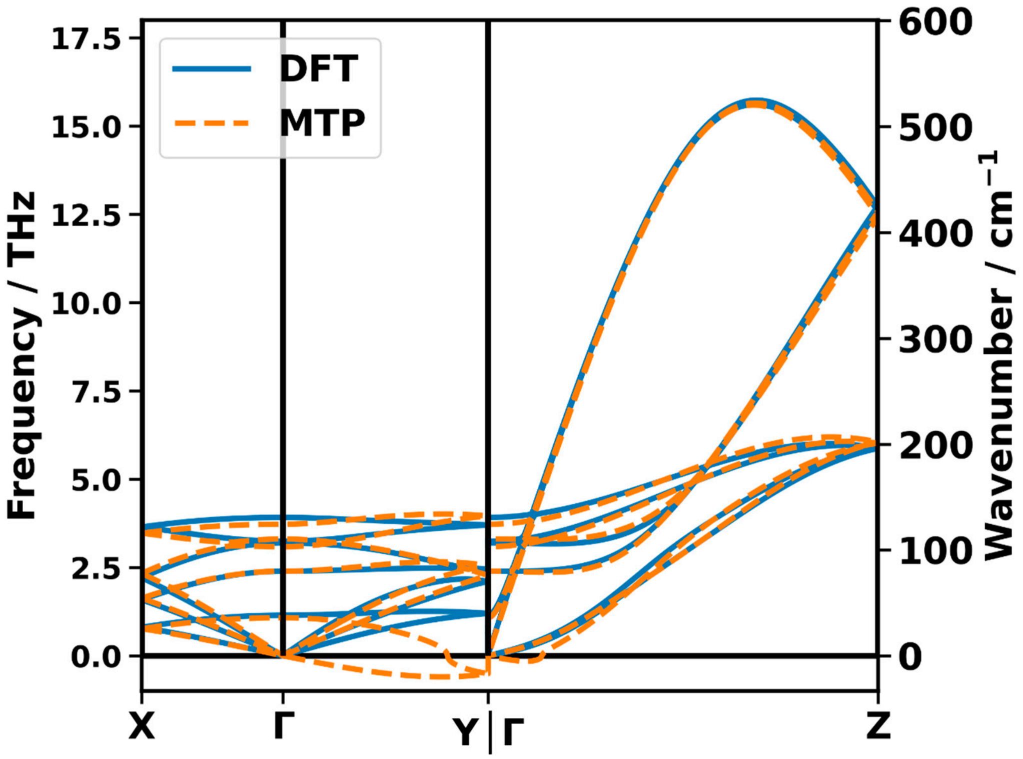

Figure S13. *Phonon band structure of PE calculated with DFT (solid blue line) and the "best"* MTP^{MD} *(dashed orange line) in the low frequency region.*

S21 Phonon Band Structures with MTP-Relaxed Unit Cell

In the main paper, band structures were calculated using MTPs with DFT-relaxed unit cells. Here, we use the same MTPs, but employ MTP-relaxed unit cells instead. For this, the "best" MTPs are used, which are defined in the main paper. Table S16 lists the $RMSD^{phonon}$ of MTPs with DFT-relaxed cells (identical to the main paper) alongside the corresponding ones with MTP-relaxed unit cells. Overall, the impact of the unit cell relaxation is minor for PE, which is consistent with the observation that the MTPs of PE can describe the unit cell very well. For P3HT, the influence of the MTP relaxation is moderate. For PT, the unit cell relaxation with the MTP notably changes the $RMSD^{phonon}$ — especially in the case of the MTP^{phonon}. Here, the unit cell optimization results in more than a tripling of $RMSD^{phonon}$. To shed light on this rather large increase in $RMSD^{phonon}$, the phonon band structures are shown employing the DFT-relaxed unit cell,

as well as the MTP-relaxed unit cell in Figure S14. Evidently, both band structures look very similar, because even though the $RMSD^{phonon}$ more than triples, it is still very low, leading to only slight changes in the overall phonon band structure. The band structure employing the MTP-relaxed unit cell is shifted up in frequency, whereas the slopes of the bands are mostly the same for both unit cells. Overall, the effect that using the MTP-relaxed unit cell instead of the DFT-relaxed unit cell has on the band structure is very small, because the MTPs predict unit cells that are very close to the DFT-relaxed ones.

Table S16. *$RMSD^{phonon}$ with the DFT-relaxed unit cell and the MTP-relaxed unit cell for different MTPs. For each material and type of MTP, we took the MTP with the lowest $RMSD^{phonon}$ (calculated for the DFT-relaxed unit cell). For each pair of calculations with the DFT-relaxed and MTP-relaxed unit cell, the same MTP was used.*

	PE [THz]	PT [THz]	P3HT [THz]
**MTP^{phonon}, DFT cell**	0.043	0.029	0.036
**MTP^{phonon}, MTP cell**	0.053	0.094	0.046
**MTP^{MD}, DFT cell**	0.152	0.080	0.059
**MTP^{MD}, MTP cell**	0.163	0.125	0.072

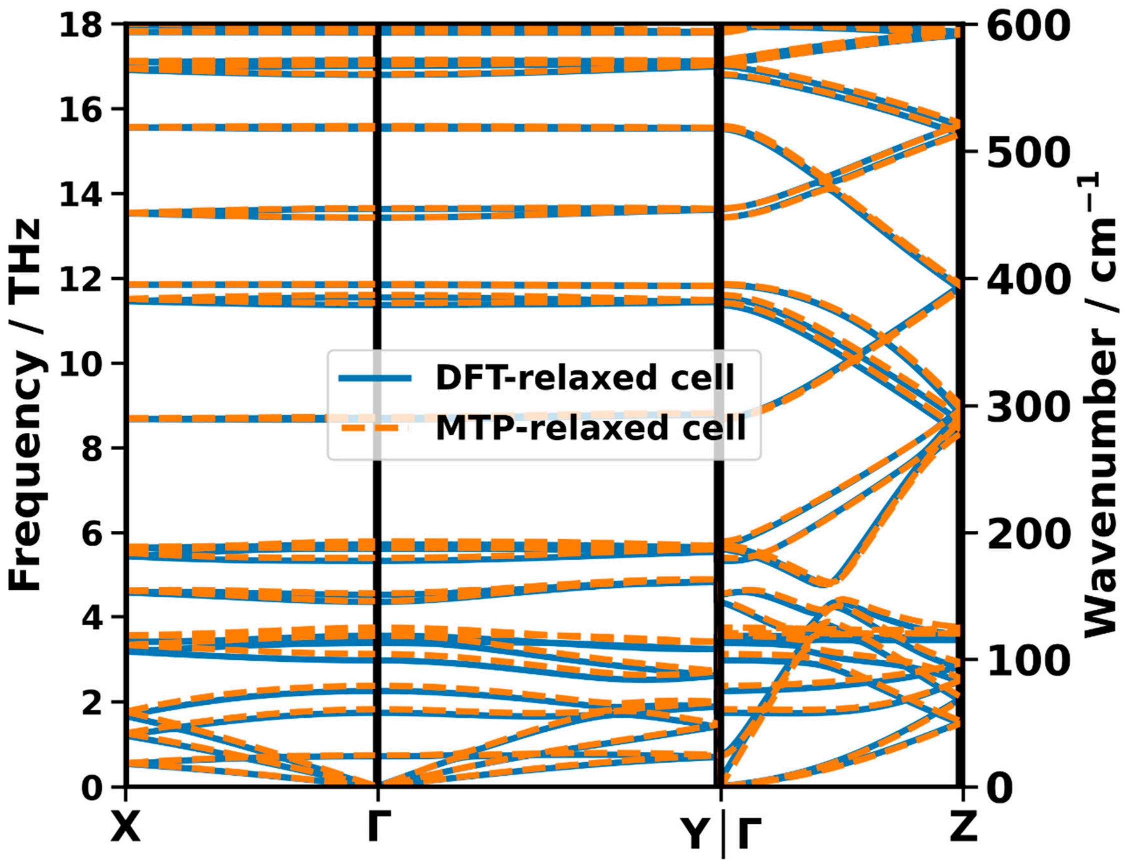

Figure S14. *Phonon band structure of PT employing the DFT-relaxed and MTP-relaxed unit cells (which results in marginally shifted x-scales in the plot). For both calculations, we used the "best" MTP^{phonon}, as defined in the main paper. The $RMSD^{phonon}$ with the DFT-relaxed unit cell is 0.029 THz and the $RMSD^{phonon}$ with the MTP-relaxed unit cell is 0.094 THz.*

S22 Phonon Density of States Over the Full Frequency Range

Phonon band structures are shown only in the low-frequency regime for reasons of visibility, typically with excellent agreement between MTP and DFT, as demonstrated in the main paper. Generally, the agreement is of the same quality also for the higher frequencies. To show this, the phonon density of states (DOS) is presented here, as it can be plotted in a compact way over the entire frequency range in one figure per material. The DOSs of PE, PT, and P3HT are shown in Figure S15, Figure S16, and Figure S17, respectively. For all three materials, the agreement between DFT and MTP is excellent across the entire frequency range. For the calculation of the DOS, we used the same MTPs that were used to calculate the phonon band structure in the main paper. These are the MTPs^{phonon} with the best RMSDs^{phonon}. The DOSs are calculated with the phonopy API in Python [9,81]. They are smeared by 0.2 THz with a Gaussian function. The "frequency pitch" controls at what interval the DOS is computed and it is fixed to 0.01 THz. The mesh for Brillouin zone integration was checked for convergence for each material individually and it is 32×48×96, 32×32×48, and 16×8×16 for PE, PT, and P3HT, respectively.

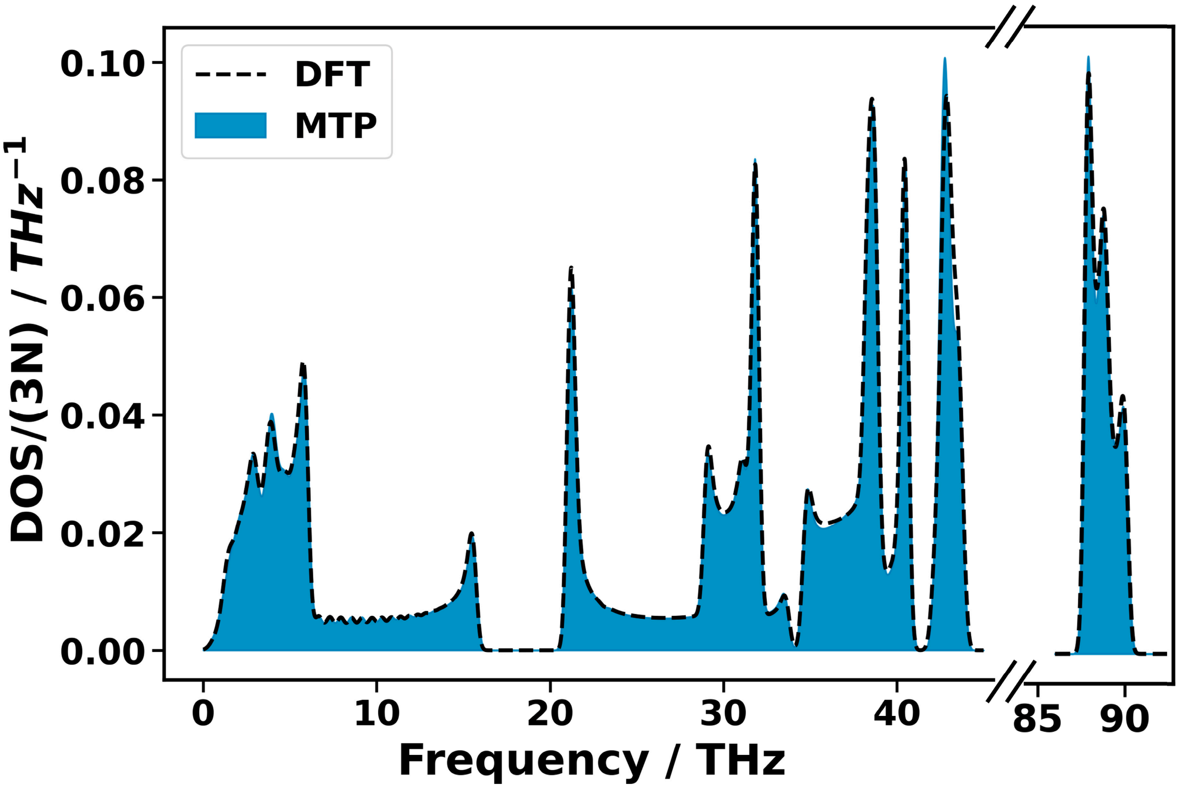

Figure S15. *Phonon density of stated (DOS) of PE calculated with DFT and the "best" MTP^{phonon}. The DOS is divided by three times the number of atoms in the unit cell (3N).*

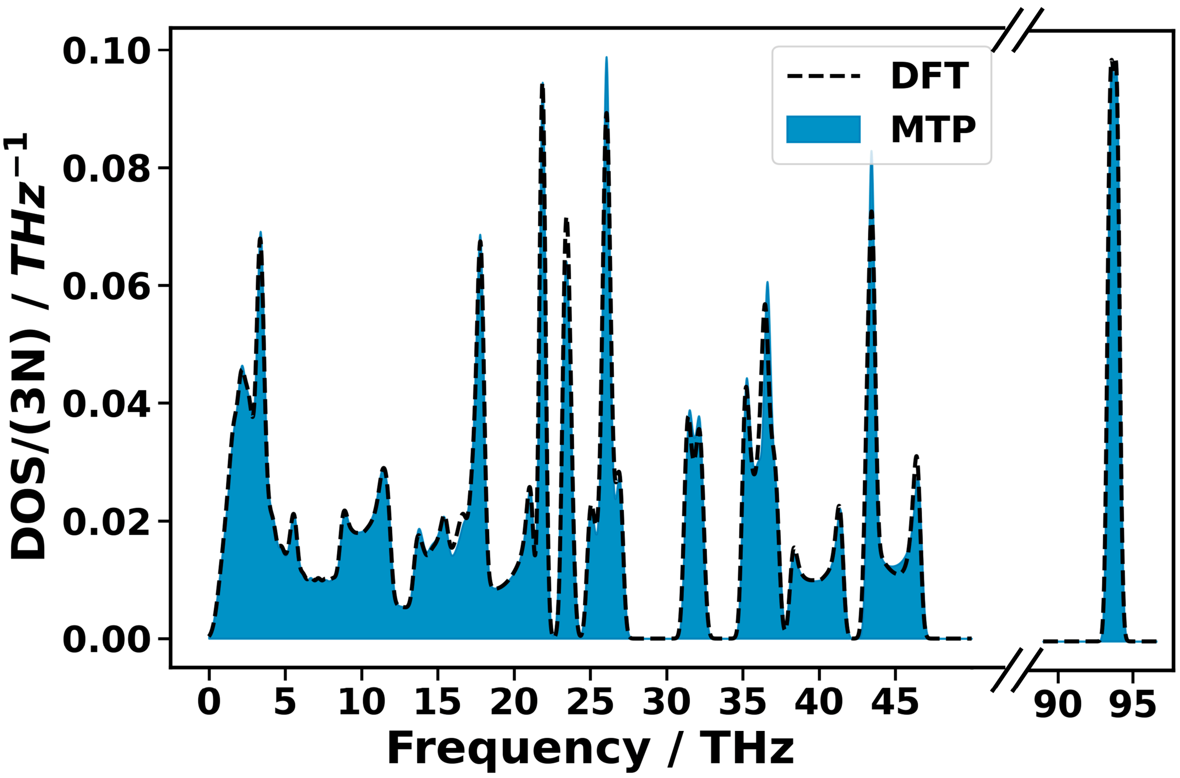

Figure S16. *Phonon density of stated (DOS) of PT calculated with DFT and the "best" MTP^{phonon}. The DOS is divided by three times the number of atoms in the unit cell (3N).*

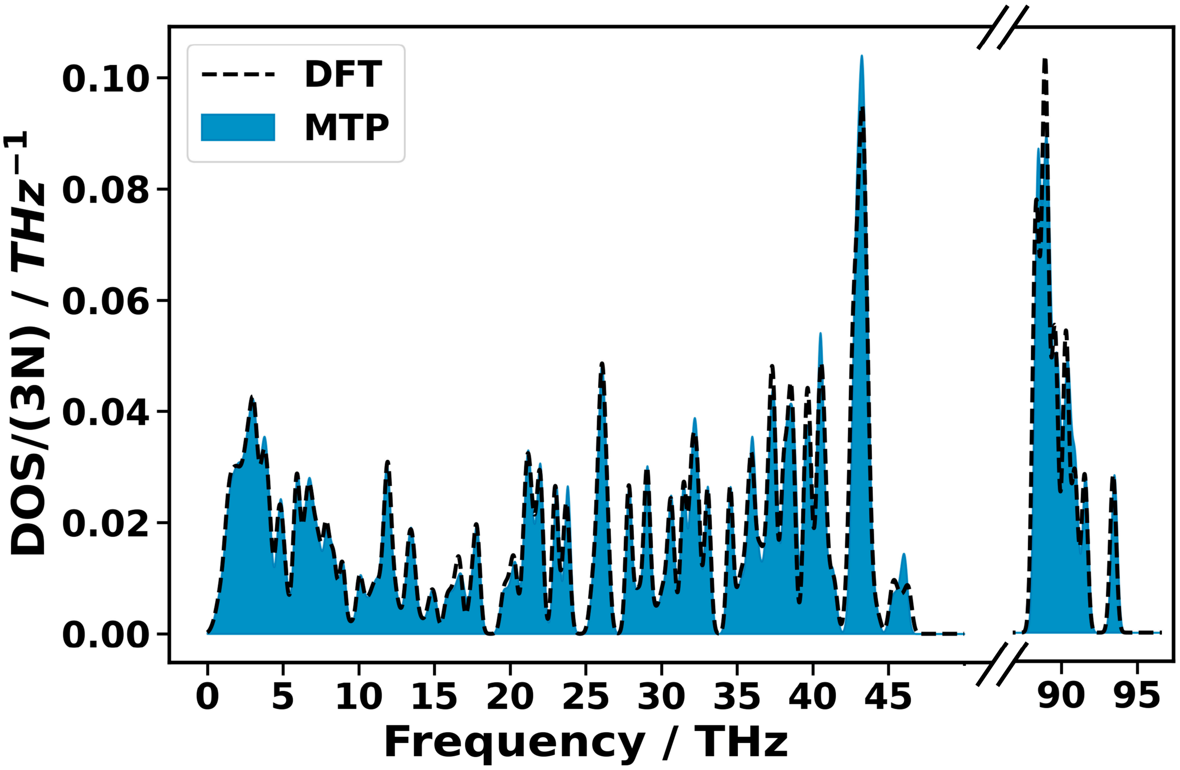

Figure S17. *Phonon density of stated (DOS) of P3HT calculated with DFT and the "best" MTP^{phonon}. The DOS is divided by three times the number of atoms in the unit cell (3N).*

S23 Functional Form of the AIREBO Potential

The functional form of the AIREBO potential is given by

$$E = \frac{1}{2}\sum_{i}\sum_{j\neq i}\left[E_{ij}^{REBO} + E_{ij}^{LJ} + \sum_{k\neq i}\sum_{l\neq i,j,k} E_{kijl}^{TORSION}\right]$$

Where the E_{ij}^{REBO} term describes short-ranged C-C, C-H, and H-H interactions and is the same as in [98]. The E_{ij}^{LJ} term accounts for longer-ranged interactions using a similar form as the standard Lennard–Jones potential. Finally, the $E_{kijl}^{TORSION}$ term is an explicit 4-body potential describing dihedral angle preferences in hydrocarbon configurations. More details on the AIREBO potential can be found in [71].

S24 Thermal Expansion Calculated with each of the Five MTPs^{MD} of PE

As stated repeatedly, five MTPs^{MD} were trained with different random initializations. For each of them, we performed MD simulations at different temperatures to determine the thermal expansion of PE. The mean and standard deviation of the thermal expansion were calculated for the lattice parameters obtained with these five MTPs and are reported in the main paper in Figure 10. To complement this plot, we here show the results for each of the five MTPs individually (Figure S18).

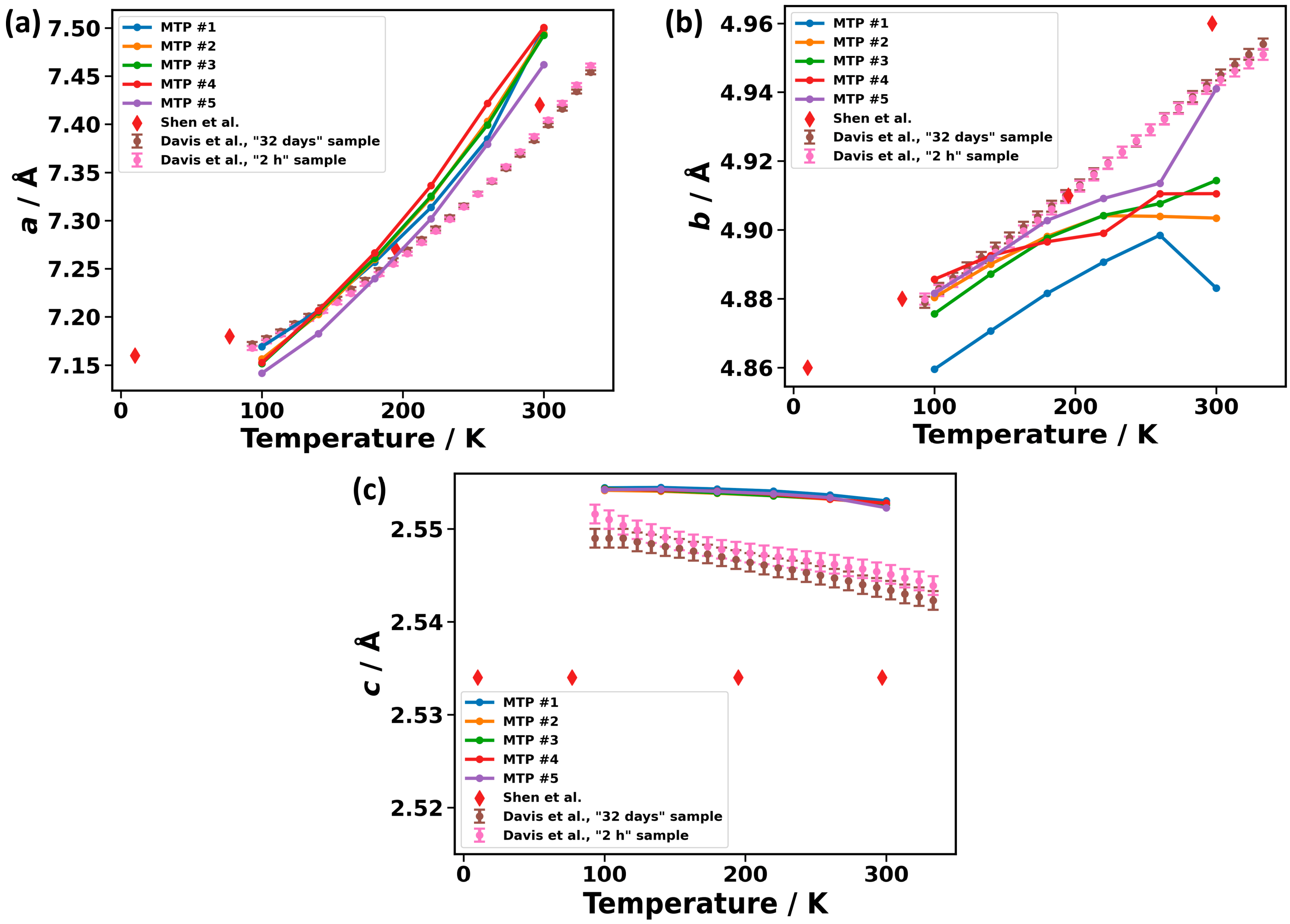

Figure S18. Thermal expansion of PE along its lattice vectors. Similar to Figure 10 in the main paper, but here each of the five MTPs are plotted individually. They are labelled with "MTP #1" to "MTP #5". Experimental data by Shen et al. and Davis et al. are shown [55,78]. Davis measured two samples, which they called "32 days" and "2 h". These names refer to how long the samples remained in constant temperature baths during their production.

S25 Thermal Expansion with Level 18 MTP

To investigate the influence of the level on thermal expansion, five MTPs with level 18 were parametrized in the same fashion as the level 22 MTPs. A comparison of the mean lattice parameters obtained with the level 18 and level 22 MTPs is shown in Figure S19. The level 22 MTPs are identical to the ones presented in the main paper. There, we showed that the level 18 MTPs are less accurate in terms of their $RMSD^{phonon}$ and $RMSD^{MD}$. This decreased accuracy seems to also be reflected here, as the level 18 MTPs show a larger spread (i.e., standard deviation plotted as error bar) than the level 22 MTPs. Still, they produce a reasonable thermal expansion with correct trends. We conclude that level 18 MTPs can be used as a cheaper alternative to level 22 MTPs, if one is willing to sacrifice some accuracy.

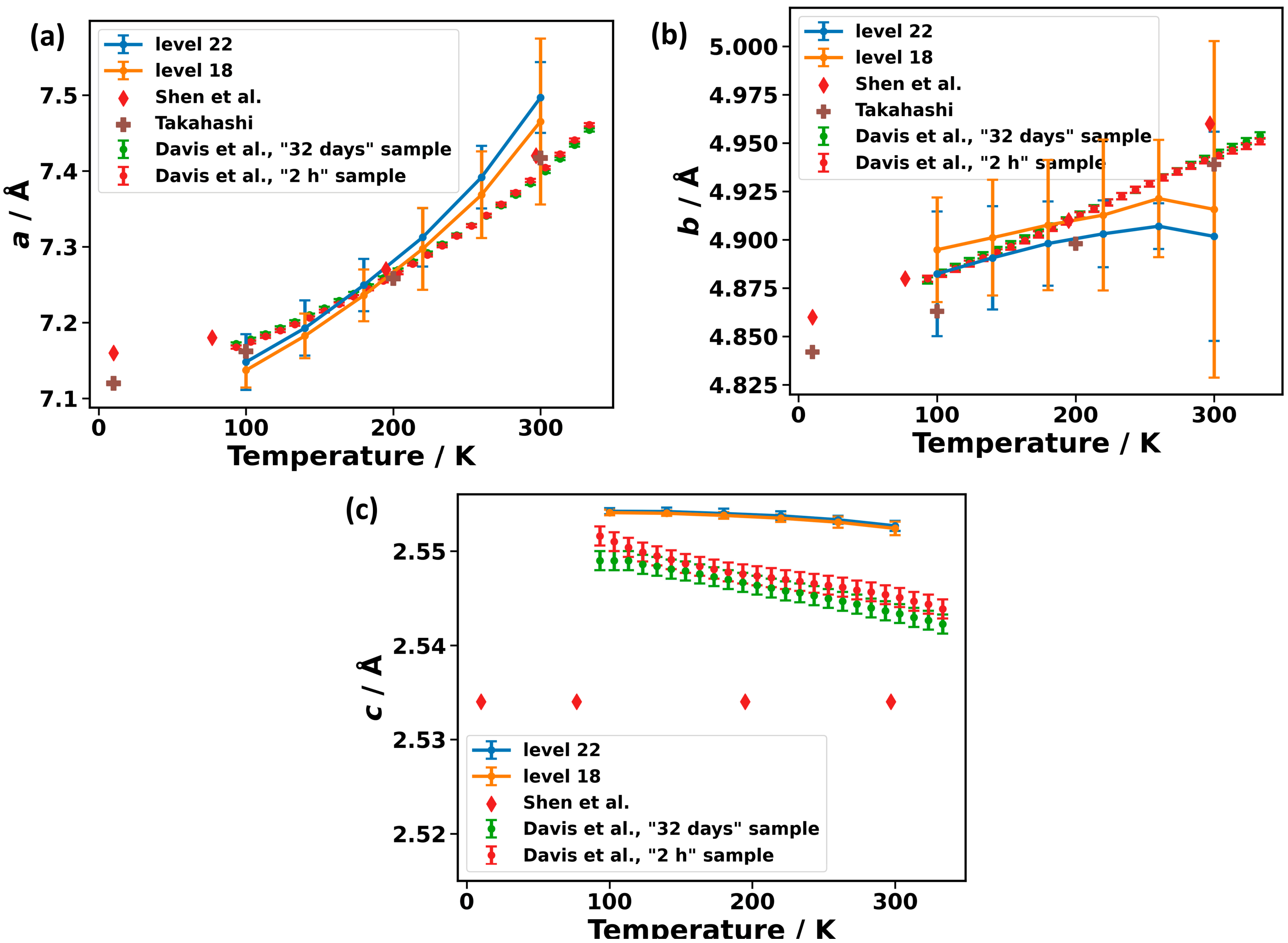

Figure S19. Thermal expansion of PE with level 22 and level 18 MTPs. For each level, five MTPs were parametrized, and the mean and standard deviation were calculated over the resulting lattice constants. The error bars of "level 22" and "level 18" are these standard deviations. As in the main paper, experimental data by Shen et al. [55], Davis et al. [78] and Takahashi [53] are shown,.

S26 Thermal Expansion Calculated with the AIREBO Potential

To put the accuracy of the MTPs for calculating thermal expansion into perspective, they are compared to the AIREBO potential. The thermal expansion calculated with the AIREBO potential is shown in Figure S20. For the most part, lattice parameters calculated with the AIREBEO potential deviate significantly from experiments. Lattice vectors ***a*** and ***b*** are both shifted by around 0.1 Å when comparing the AIREBO potential to experiments by Shen et al. and Davis et al. [55,78]. However, the slope with temperature fits rather well to the experiments. This means that the thermal expansion coefficient, which is the derivative of the lattice parameters with respect to temperature, is reasonably well described by the AIREBO potential. The AIREBO potential predicts a wrong trend only for the lattice vector ***c***. That is, it predicts positive thermal expansion, whereas it is negative in the experiment by Davis et al. [78]. Here, one, however, has to keep in mind that the changes in ***c*** with temperature are marginal. The good agreement of the thermal expansion in ***a***- and ***b***-direction (vdW-bonded directions) could be due to the Lennard–Jones term in the AIREBO potential, which, in our experience, works rather well for van der Waals bonded organic materials, as explicitly shown, for example, for naphthalene (where the also rather simple generalized AMBER force field [99] describes acoustic phonons well [17]).

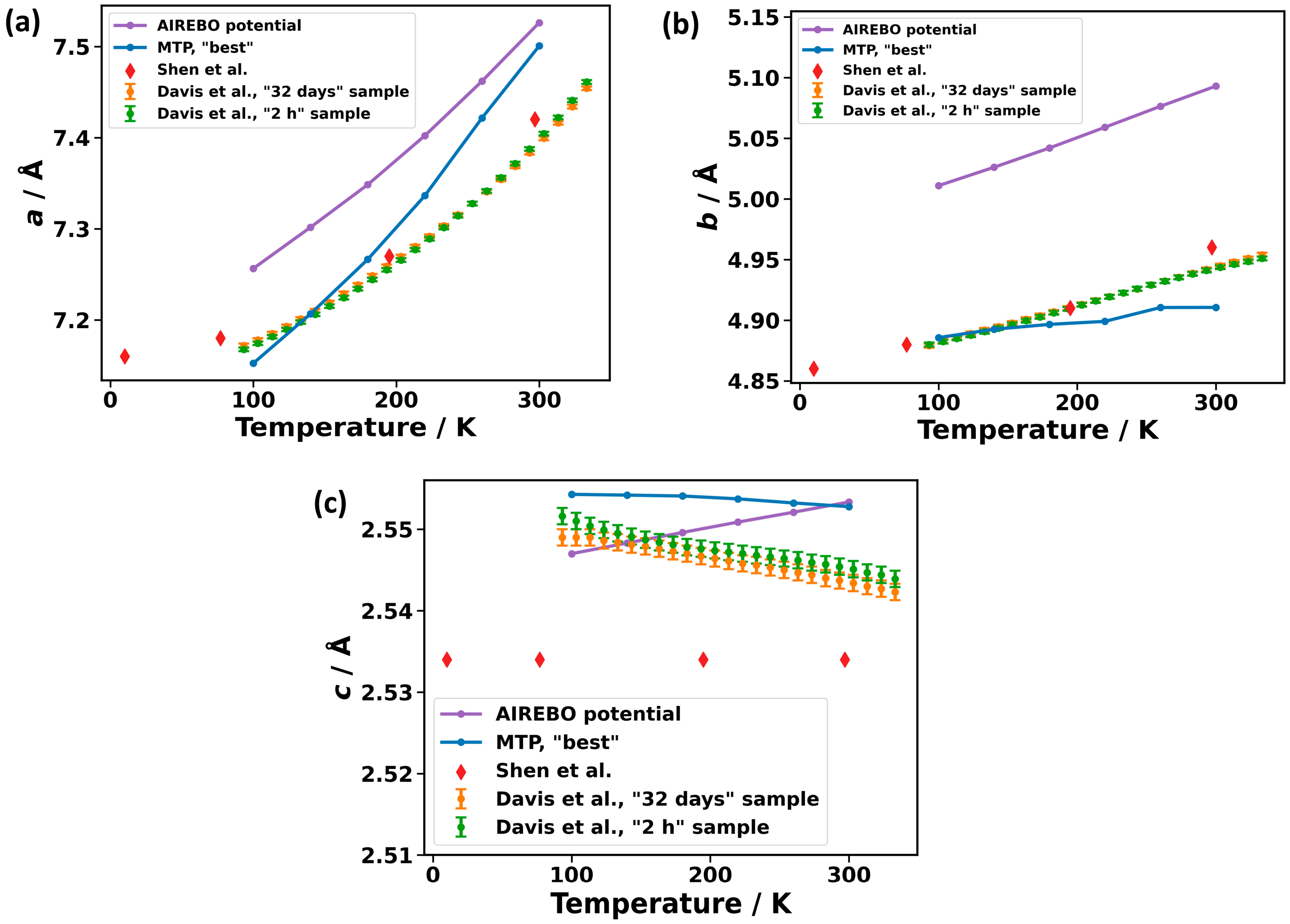

Figure S20. Thermal expansion of PE along its lattice vectors (a) ***a****, (b)* ***b****, and (c)* ***c****. Similar to Figure 10 from the main manuscript, but here also with data calculated using the AIREBO potential. The experimental data by Shen et al. and Davis et al. are also shown [55,78].*

S27 Details on Computational Efficiency of MTPs and DFT

In the main text, we compared the speed of MTPs with DFT and made the rough estimation that MTPs are 10^5 times faster than DFT for the phonon calculations of PE, which were performed with 432 atoms in the supercell. In the following, we will give more computational details on these calculations. Calculations were performed on the Vienna Scientific Cluster 5 (VSC-5), which has 2 AMD EPYC 7713 (each with 64 cores) per node. On this cluster, a DFT single point calculation with VASP takes 715 s for the 432-atom supercell of PE. Evaluating the duration of a single force calculation for this cell with MTP^{phonon} would be difficult, as it only takes a fraction of a second, and the startup time of the program would tamper with our estimation of the speed. To mitigate this, we performed an MD run for 120,000 steps instead. From this we evaluate the time it takes to perform one MD step to be 0.005 s. This calculation was performed with the MTP^{MD} (level 22), which is ~2.25 times faster than the MTP^{phonon} (level 26). Dividing 715 s by 0.005 s, we obtain as a result that MTP^{MD} is ~143,000 times faster than DFT and MTP^{phonon} is ~64,000 times faster than DFT. Thus, we report 10^5 as an order of magnitude estimate for the speedup.

In the main paper, we also commented on the time it takes to parametrize the MTPs. For the MTP^{phonon} of PE, we parametrized five MTPs which took 28 h, 23 h, 25h, 20h, and 20h on a single node of the VSC-5. This computes to an average training time of 23 h. Dividing 23 h by the aforementioned 715 s, we can say that the training time of a MTP^{phonon} is equivalent to the time it takes to perform 116 singe point calculations with DFT. For MTP^{MD}, there are two competing differences with MTP^{phonon}: On the one hand, the training time is prolonged by having more training data, but, on the other hand, it is shortened by having a lower level. The latter effect dominates leading to an overall shortening of the average training time of MTP^{MD} to 16 h, which requires as much computation as 81 DFT calculations. Again, we want to emphasize that these numbers depend on the used hardware, the number of atoms, the resulting parallelization, and the material of interest. Therefore, the reported 10^5 times speedup should be understood as an order of magnitude estimate.

S28 Details on Computational Efficiency Comparison of MTPs with different Levels

In section 2.2 of the main text, a comparison between levels 18, 22, and 26 was performed in terms of their accuracy. Here, we want to compare the speeds of these MTPs with different levels. The calculations were performed on the VSC-5 (see above). The MTPs follow the MTP^{phonon} paradigm, which means that they are trained on data sampled at 15 K to 100 K. Since the system chosen for this test is PE, no atom typing is performed. To test the speed, molecular dynamics runs with 50,000 time steps were performed at 30 K. The supercell size is 4×6×12, which is the same size that was used for the thermal expansion calculations. The computation time as reported by LAMMPS is 533 s, 1231 s, and 2486 s for levels 18, 22, and 26, respectively. Hence, in this particular case, the level 18 MTP is 2.3 times faster than the level 22 MTP and 4.7 times faster than the level 26 MTP. In general, we found that, as a rule of thumb, increasing the level by 2 increases the computational demands by roughly a factor of 1.5, which is also consistent with the example mentioned here.

S29 Convergence Tests

In this section, various convergence tests are shown.

S29.1 Energy Cutoff

As a first strategy to estimate which plane wave energy cutoff and $\boldsymbol{k}$-mesh would be suitable for the DFT simulations, we checked that the total energy per atom should be converged to below 0.5 meV. This procedure suggested an energy cutoff of 900 eV for PE, which is, in our experience, often adequate for calculating phonon properties. Of course, this only serves as a first guess, as one should check convergence on the quantity of interest (like the phonon band structure) explicitly. In Figure S21, one can see that the phonon band structure of PE calculated with 700 eV and 900 eV is almost indistinguishable, supporting the notion that an energy cutoff of 900 eV (or even 700 eV for that matter) is sufficient. Thus, for PE, an energy cutoff of 900 eV was used throughout this work, except for the unit cell relaxation, where it is increased to 1350 eV, and except for the BTE calculation, where it was decreased to 700 eV. This was performed to save computational resources considering the enormous computational effort of calculating third-order force constants and in view of the observation in Figure S21 that the phonon band structure is already essentially converged for a 700 eV

cutoff. Analogously, for PT and P3HT, phonon band structures with an energy cutoff of 700 eV and 900 eV were calculated, and they were again identical within a small margin.

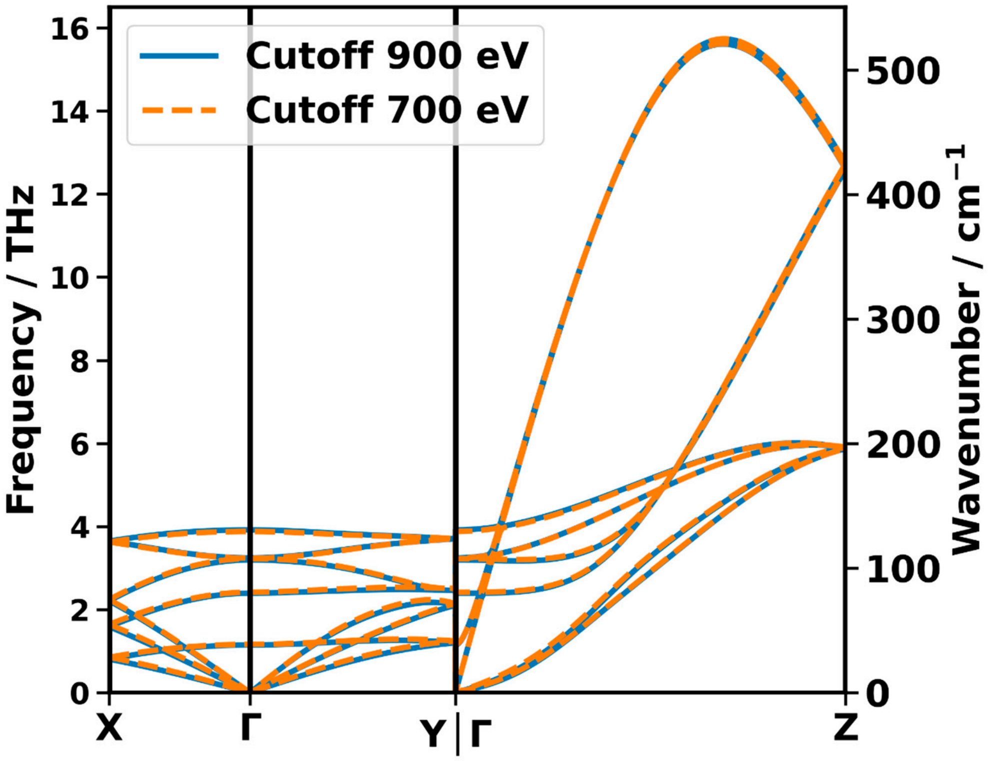

Figure S21. *Phonon band structure of PE calculated with DFT with an energy cutoff of 700 eV and 900 eV, which define the used basis set. A 2×3×6 supercell is used and the* ***k****-mesh in the electronic calculations for that supercell includes only the Γ-point.*

S29.2 K-Mesh for DFT

In the same fashion as for the energy cutoff, different ***k***-meshes were tested by performing phonon band structure calculations with DFT. Figure S22 shows that a ***k***-mesh for the electronic calculation of 1×1×1 (i.e., just the Γ-point) and a 2×2×2 ***k***-mesh produce phonon band structures that only deviate marginally from each other. Therefore, a 1×1×1 ***k***-mesh is sufficient for PE with a 2×3×6 supercell. For PT, we found from the convergence tests on the energy (as explained in Section S29.1) that an additional ***k***-point along the chain direction is necessary, which is consistent with the much larger band dispersion along the chain direction in that semiconducting material. As far as PT's phonon band structure is concerned, it hardly changes when switching from a 1×1×2 to a 2×2×4 ***k***-mesh, except for the third lowest band at the Y-point, whose position differs by 0.1 THz. For PE, we also checked the convergence of the MTPs with respect to the energy cutoff and ***k***-mesh by training it on DFT reference calculated data with an energy cutoff 1100 and 2×2×2 ***k***-mesh. This does not yield any significant differences in band structures calculated with these MTPs.

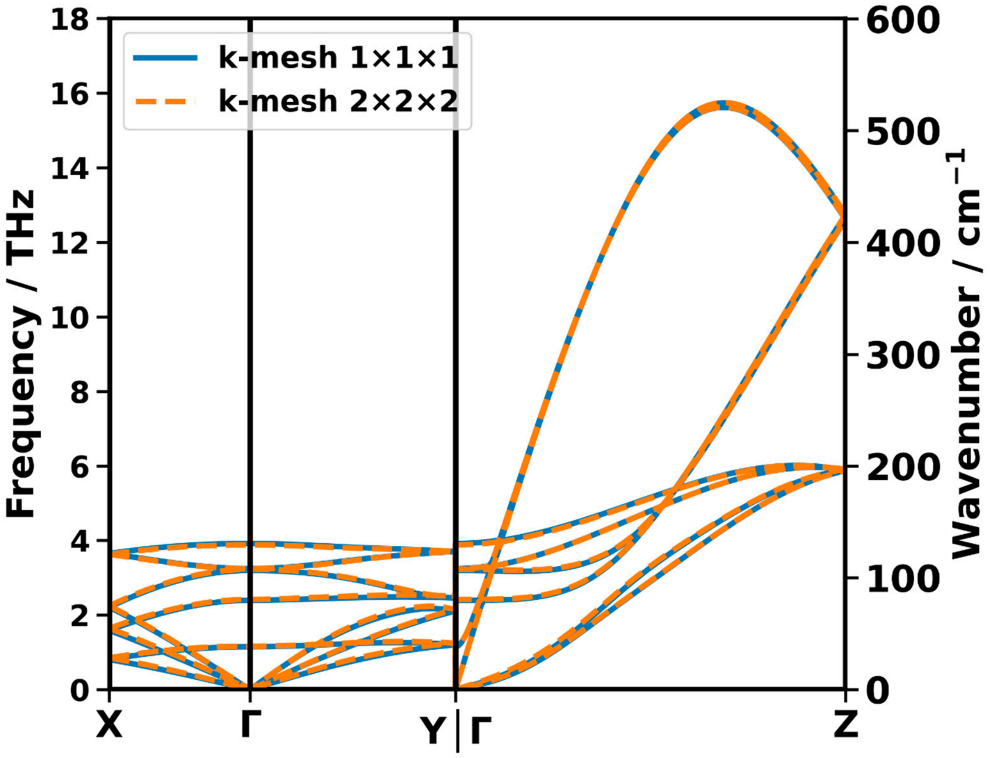

Figure S22. *Phonon band structure of PE calculated with DFT with a* ***k****-mesh of 1×1×1 and 2×2×2. The supercell is 2×3×6 times the primitive cell and the energy cutoff is fixed to 900 eV.*

S29.3 Strain Distance for the Calculation of Elastic Constants

The elastic stiffness tensor elements (for short: elastic constants) are calculated via a finite difference approach (called "clamped ion method"), whereby a small strain distance is used, which needs to be converged. To that end, Figures S23-S25 show the elastic constants calculated for different strain distances. These figures show the results with DFT, MTP^{phonon}, and MTP^{MD}. Focusing first on the DFT calculations (Figure S23), for PE, all shown strain distances of 1 %, 0.1 %, and 0.01% give very similar values. For PT and P3HT, the situation is slightly different, as a strain distance of 0.01 % gives elastic constants that deviate from the ones calculated with 0.1 % and 1 %. Therefore, we regard 0.01 % as a too small strain distance for PT and P3HT. Thus, strain distances of 0.1 % and 1 % can be considered as converged with DFT, as they give both very similar values for all materials. For MTP^{phonon}, all three strains give very similar elastic constants for all materials. For MTP^{MD}, the situation is slightly more involved. For PE with MTP^{MD}, the smallest strain of 0.01 % gives deviating elastic constants. For P3HT with MTP^{MD}, the C_{23} element at 1 % strain shows a deviation as compared to the other strains. We note, however, that this seeming deviation is mostly a consequence of the logarithmic plotting. C_{23} is 1.67 GPa, 1.43 GPa, and 1.40 GPa for strains of 1 %, 0.1 %, and 0.01 %, respectively. Aside from this very particular element, the strain of 1 % gives the best convergence overall. Therefore, we eventually opted for 1 % as the strain distance, that was used for the calculations in the main paper.

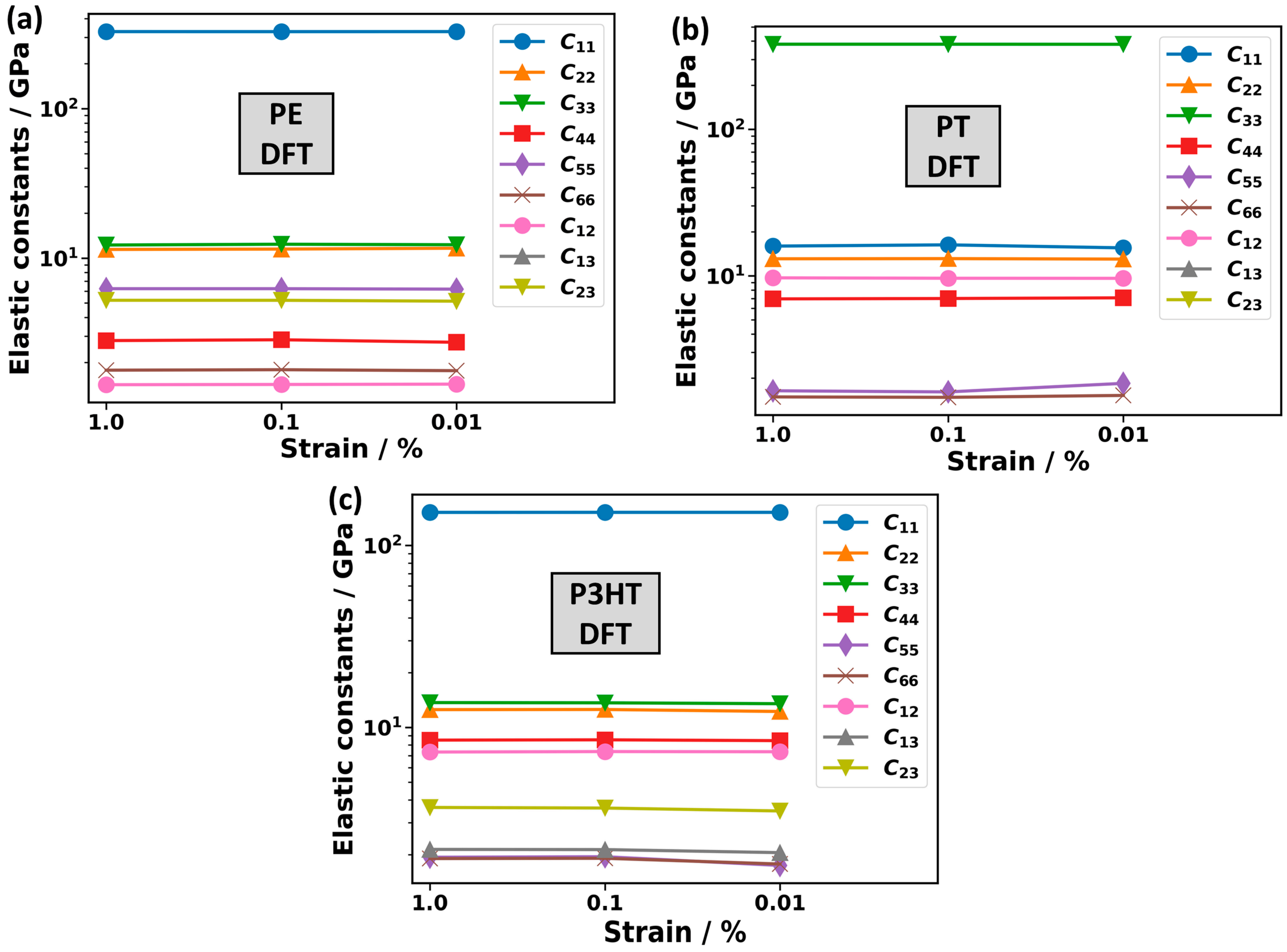

Figure S23. *Elastic constants calculated with DFT for different strains of the unit cell. The materials are (a) PE, (b) PT, and (c) P3HT.*

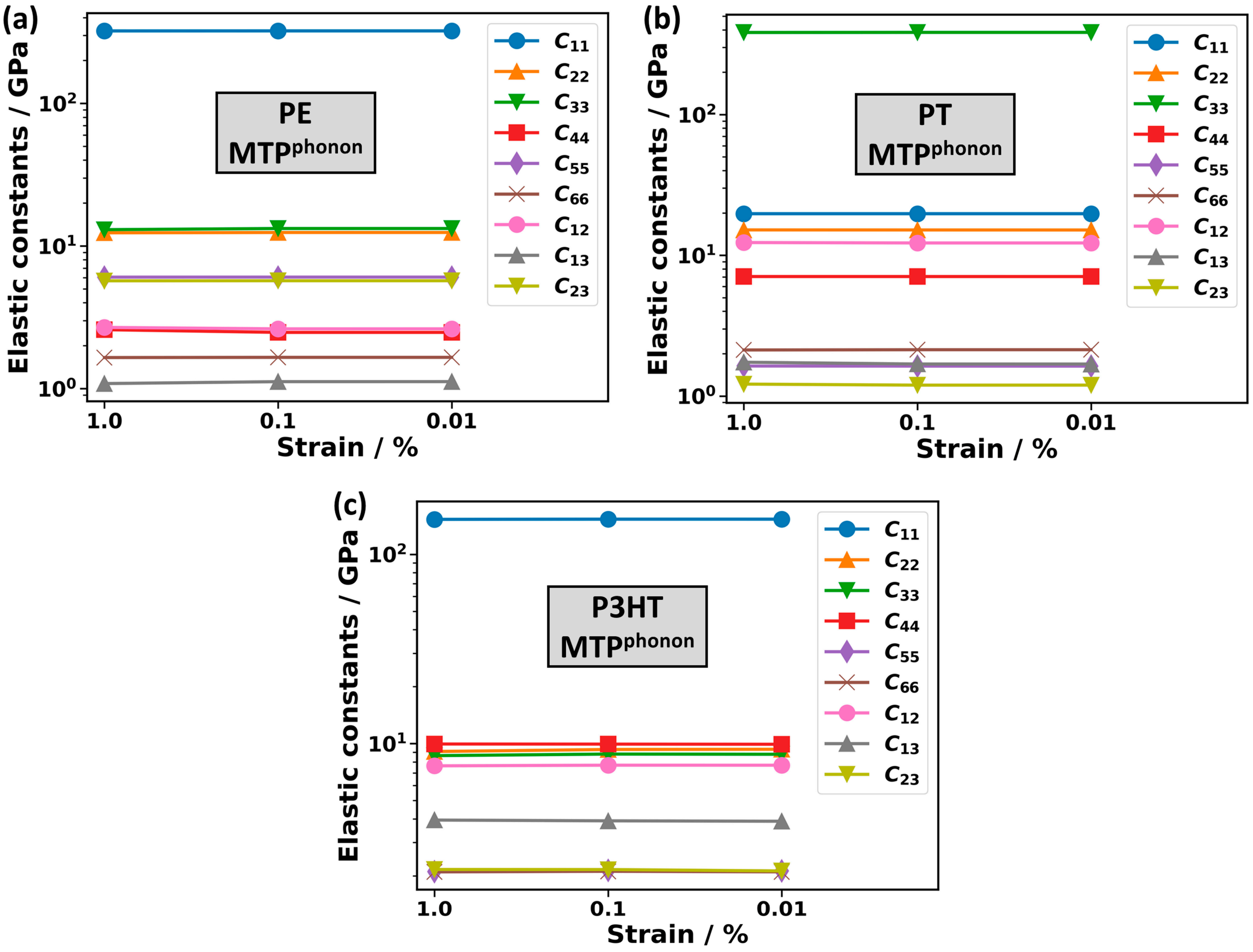

Figure S24. *Elastic constants calculated with MTP*phonon *for different strains of the unit cell. The materials are (a) PE, (b) PT, and (c) P3HT.*

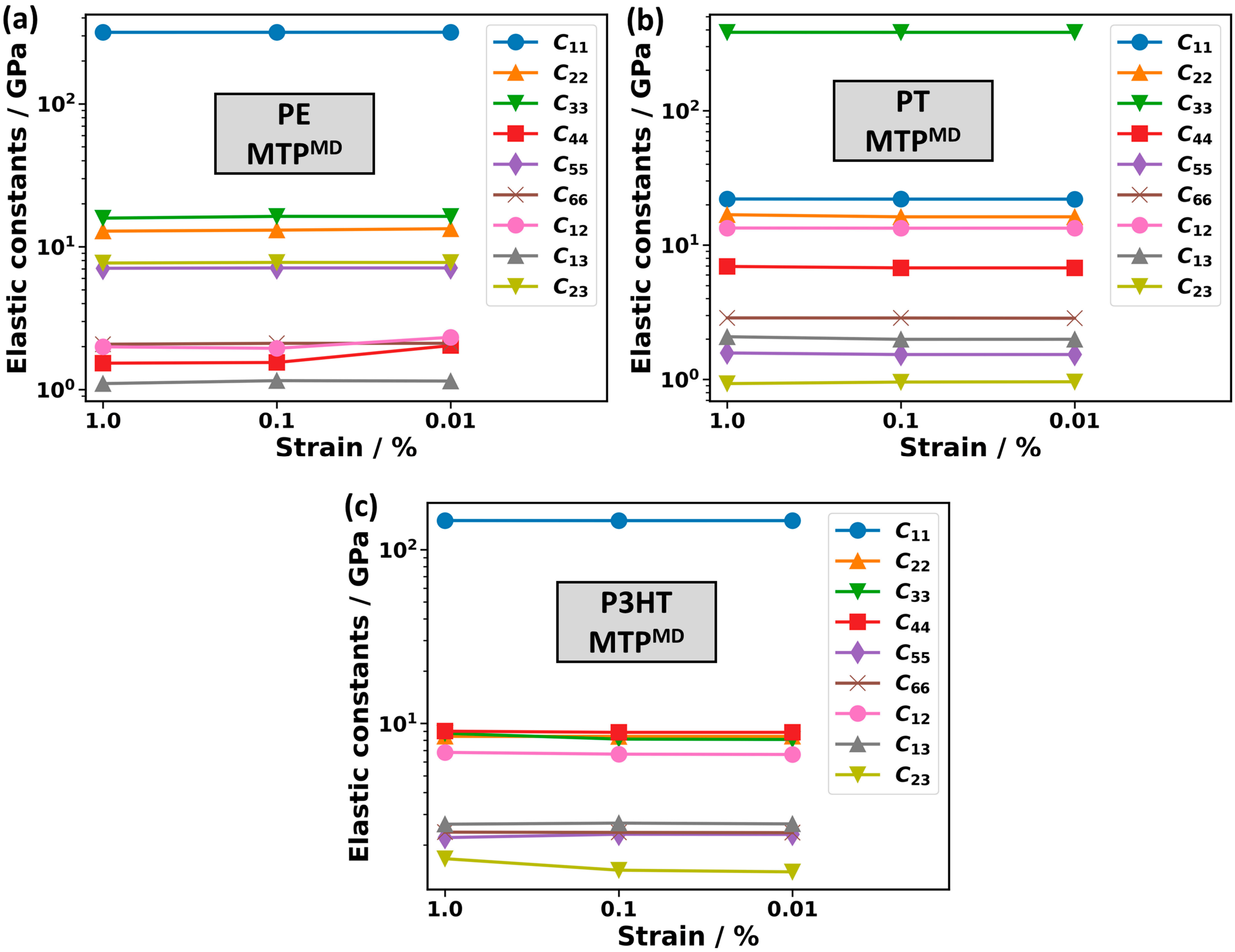

Figure S25. Elastic constants calculated with MTP^{MD} for different strains of the unit cell. The materials are (a) PE, (b) PT, and (c) P3HT.

S29.4 Supercell Convergence for Calculating Second-Order Force Constants

One has to converge the supercell size for the calculation of the second-order force constants. As a rule of thumb, we found that the phonon band structure is typically converged for supercells with an extent of about 15 Å in each direction. This rule of thumb can be used as a starting point, from which we explicitly checked the convergence for each material. For PE, the 2×3×6 supercell is approximately 15 Å long in each direction. We explicitly checked the convergence by calculating the phonon band structure with a 2×3×9 and a 2×5×6 supercell and comparing the results with a 2×3×6 supercell. A comparison between the 2×3×6 and 2×3×9 supercells is shown in Figure S26. The two band structures are essentially identical, thus showing that the supercell 2×3×6 is converged. Similarly, the phonon band structures calculated with a 2×3×6 and 2×5×6 supercell are also essentially identical. Analogous convergence tests were performed for PT and P3HT yielding supercell convergence for 2×3×4 and 2×1×2 cells.

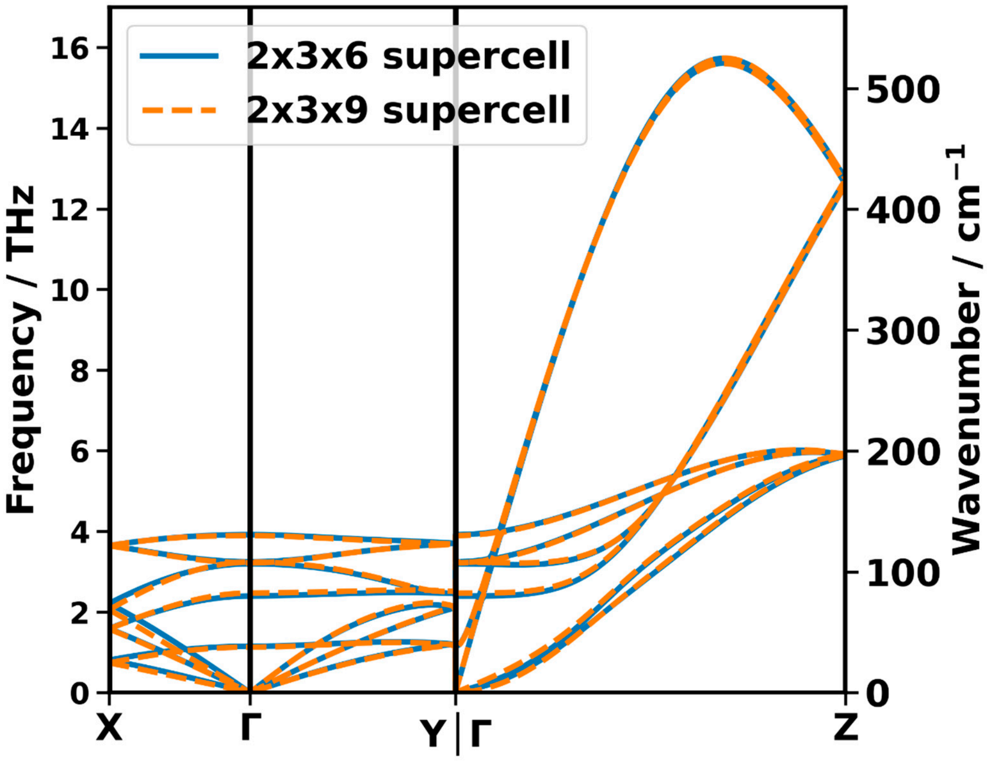

Figure S26. *Phonon band structure of PE calculated with DFT for 2×3×6 and 2×3×9 supercells.*

S29.5 Supercell Convergence for Calculating Third-Order Force Constants

In phono3py, one can choose the size of the supercell for the second- and third-order force constants independently. Converging them independently is highly beneficial in terms of computational efficiency, as the supercell size for the third-order force constants greatly influences the computational effort. With DFT, it is not feasible to calculate supercells that are larger than 2×2×3 for the third-order force constants, because the calculation of any larger supercell would exceed the computational resources available to us. This 2×2×3 super cell is the supercell size that also Wang et al. chose [73]. They argue that the third-order force constants are converged for that supercell, but do not prove this explicitly. Here, we have highly efficient MTPs at our disposal, with which it is straightforward to study larger supercells. The MTP is trained on a 2×3×6 supercell, thus it is able to describe this supercell correctly. With the aid of the MTP, we can explicitly check the convergence by calculating the third-order force constants with a 2×2×3 and 2×3×6 supercell. The result is shown in Figure S27, from which we conclude that the 2×2×3 supercell provides a qualitatively correct description of third-order force constants. The thermal conductivity in the RTA is 304 W/mK and 287 W/mK for the 2×2×3 and 2×3×6 supercells, respectively. We regard this difference of 6 % as rather insignificant.

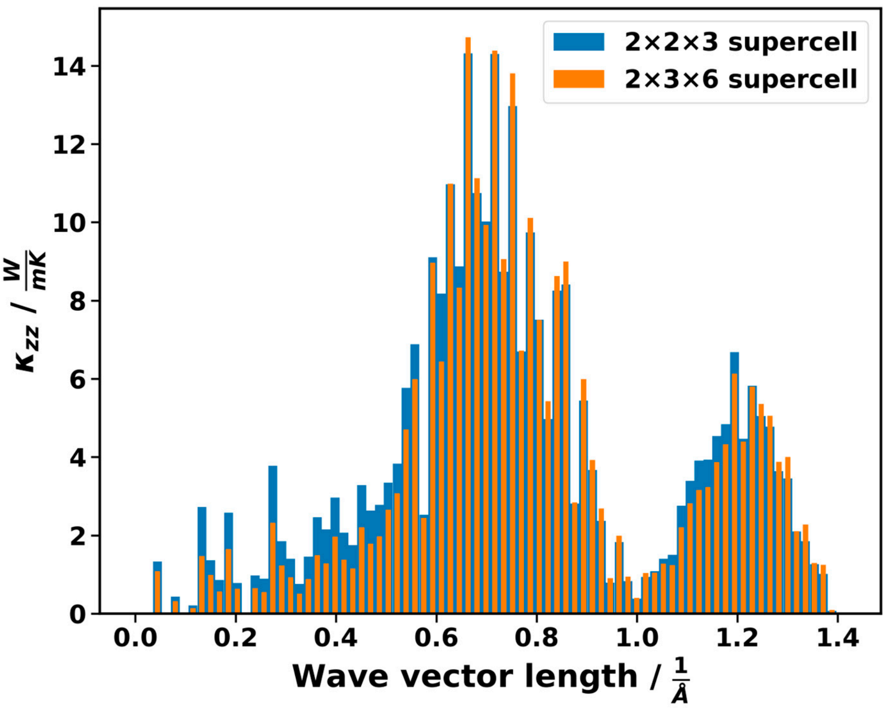

Figure S27. Mode contributions to the thermal conductivity along the chain plotted as a function of the length of the wave vector of each phonon. The same MTP and 2×3×6 supercell was used for the second-order force constants in both calculations. The only difference between the blue and orange bars is, that the third-order force constants are calculated with a 2×2×3 and 2×3×6 supercells, respectively.

S29.6 Displacement Amplitude in Phono3py

The default displacement amplitude in phono3py 2.2.0 is 0.03 Å. We tested the suitability of this amplitude by repeating the DFT calculation with 0.05 Å. In *z*-direction, the thermal conductivity is calculated to be 306.15 W/mK and 306.39 W/mK for 0.03 Å and 0.05 Å, respectively. In the ***x***- and ***y***-directions, it is 0.543 W/mK and 0.457 W/mK for an amplitude of 0.03 Å amplitude, and 0.543 W/mK and 0.460 W/mK for 0.05 Å. This excellent agreement supports the suitability of the used displacement amplitude of 0.03 Å.

S29.7 Q-Mesh for Phono3py

Following the calculation of the second- and third-order force constants, they are used to calculate phonon lifetimes and the thermal conductivity of PE. In the calculation of the thermal conductivity, the Brillouin zone is discretized with a ***q***-mesh, which needs to be converged and should not be confused with the ***k***-mesh that is used for describing the electronic structure in all DFT calculations. A priori, one might assume a uniform sampling of the Brillouin zone. However, as PE has highly anisotropic interatomic interactions, we performed independent convergence tests for the ***q***-mesh in chain direction (***c***-direction) and for the ***q***-mesh in vdW-bonded directions (***a***- and ***b***-directions), whereby the sampling in the ***a***-***b***-plane is kept uniform throughout. To perform the convergence tests, one has to set a goal accuracy up to which the thermal conductivity should be converged. In the literature, a 5-10 % accuracy is typical in that regard. For instance, 10 % were used as a convergence criterion in [100]. We chose 5 % as our convergence criterion and increased the ***q***-mesh accordingly until the criterion was met. This was performed separately for the three elements of the thermal conductivity tensor, of which we will first discuss the one in chain direction. κ_{zz} in the relaxation time approximation (RTA) is

converged to within 5 % for 6 points in the **x**-direction and 10 points in the **y**-direction. The convergence of κ_{zz} with respect to the $\boldsymbol{q}$-mesh in z-direction is shown in Figure S28. There, κ_{zz} in the RTA converges in a well-behaved manner to within 5 % for 60 $\boldsymbol{q}$-points. However, the full BTE (i.e., without RTA) shows a less clear convergence behavior. The thermal conductivity wildly fluctuates for $\boldsymbol{q}$-meshes with less than 80 $\boldsymbol{q}$-points in chain direction. Eventually, for large enough $\boldsymbol{q}$-meshes, it converges to around 400 W/mK. We found that the calculations with 30, 40, 50, and 70 $\boldsymbol{q}$-points in chain direction have a negative eigenvalue of around -0.001 in their collision matrix, whose definition can be found in the respective phono3py paper [9]. We think that this negative eigenvalue caused the oscillating convergence behavior. The negative eigenvalue could be caused by the denominators, for example, in equations A23-A26 in appendix A of [81] getting close to zero. (We thank Atsushi Togo for providing feedback on this matter.) These equations are used when integration of delta functions is performed with the tetrahedron method. If we exclude the calculations in which negative eigenvalues occur, the convergence behavior is reasonable also for the full BTE calculation. Therefore, we regard our results as valid, because the full BTE eventually converges for sufficiently large $\boldsymbol{q}$-meshes. To sum up, the $\boldsymbol{q}$-mesh that fulfils the 5 % convergence criterion is 6×10×60 for κ_{zz} in the RTA. Interestingly, this efficiently converged $\boldsymbol{q}$-mesh is highly anisotropic, as a uniform sampling of the Brillouin zone would correspond to a $\boldsymbol{q}$-mesh of for example 6×10×17.

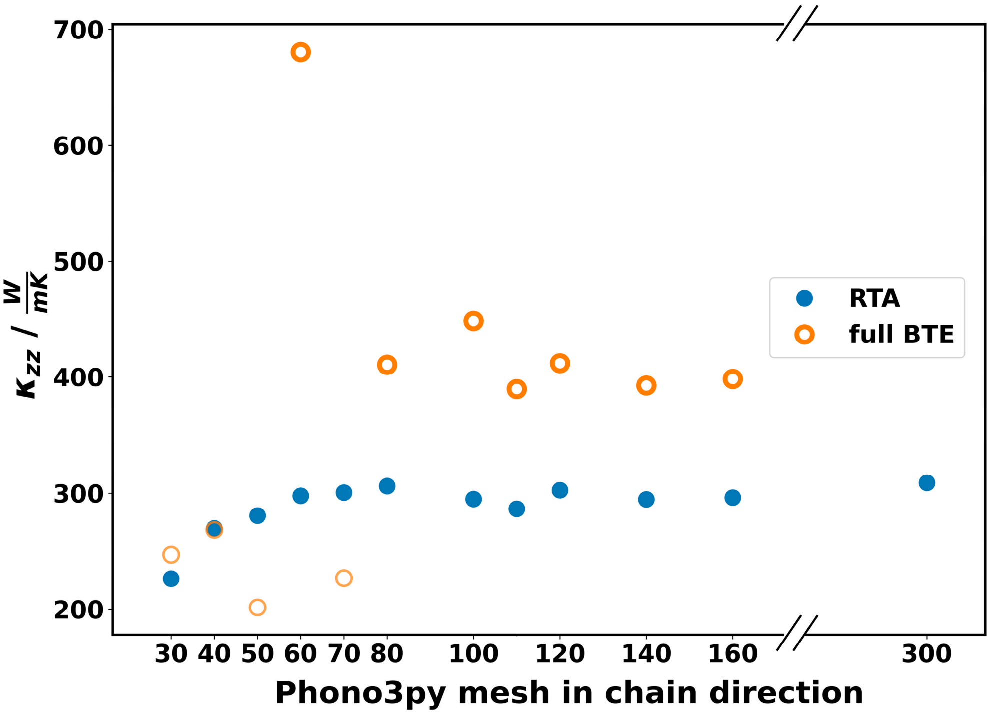

Figure S28. *The thermal conductivity of PE along the chain,* κ_{zz}*, is calculated with DFT and plotted as a function of the number of* $\boldsymbol{q}$*-points in chain direction in phono3py. Orange rings are the full BTE results, and blue dots are the results from the same calculation, but with the RTA. In the calculation with 30, 40, 50, and 50 mesh points, there were negative eigenvalues of the collision matrix. Since this causes a wrong result, these calculations are plotted semi-transparently and as thinner rings. The meshes used in these calculations are 6×10×30, 10×15×40, 10×15×50, 10×15×60, 6×10×70, 10×15×80, 6×10×100, 6×10×120, 4×6×160, and 10×15×300.*

The same procedure of finding the converged $\boldsymbol{q}$-mesh for κ_{zz} was also performed for κ_{xx} and κ_{yy}. Again, we observed that the thermal conductivity tensor elements are mostly influenced by the $\boldsymbol{q}$-point density in the direction in which the thermal conductivity is calculated. For example, already for 30 points in z-direction, κ_{yy} and κ_{yy} are

converged to below 2 % uncertainty. κ_{xx} and κ_{yy} in the RTA are converged for 6 and 10 $\boldsymbol{q}$-points in the respective directions. For the full BTE higher $\boldsymbol{q}$-meshes are necessary than for the BTE in the RTA. Namely, 10 and 15 $\boldsymbol{q}$-points in the $\boldsymbol{x}$- and $\boldsymbol{y}$-directions should be used. To sum up, a 10×15×160 $\boldsymbol{q}$-mesh would yield convergence for all thermal conductivity tensor elements of PE. However, such a calculation was infeasible due to the high memory demand. As a remedy, we split the calculation up into one sampling $\boldsymbol{q}$-space on a 10×15×60 mesh for calculating κ_{xx} and κ_{yy}, and a 4×6×160 mesh for calculating κ_{zz}.

S29.8 Time in Molecular Dynamics Calculation of Thermal Expansion

The simulation time in molecular dynamics (MD) has to be long enough such that the lattice parameters are sufficiently converged. To test this, we plot the running average of the lattice parameter ***a*** of PE in

Figure **S29**. MD was performed at 300 K with the MTP that is called "MTP #1" above. We regard the lattice length as converged after around 200 ps, because it only changes marginally for longer times. The other lattice vector lengths (***b*** and ***c***) converge faster than ***a***, presumably because ***a*** has by far the largest thermal expansion. For the calculations in the main paper, we use a somewhat over-converged time of 800 ps.

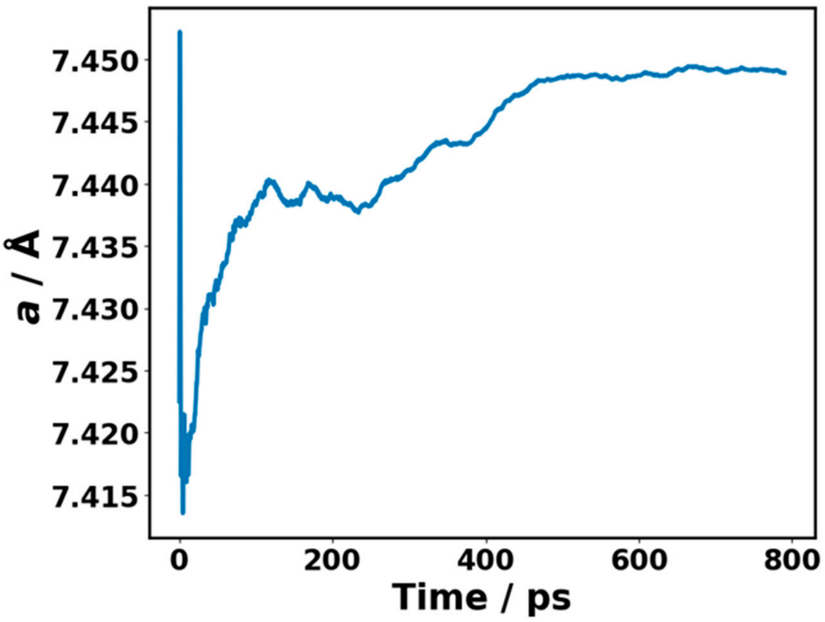

Figure S29. Running average of lattice parameter length, ***a*,** of PE during a 300 K MD run (for details see main text).

References

See main manuscript
